# The Effect of Metal Ions (Fe, Co, Ni, and Cu) on the Molecular-Structural, Protein Binding, and Cytotoxic Properties of Metal Pyridoxal-Thiosemicarbazone Complexes

**DOI:** 10.3390/ijms241511910

**Published:** 2023-07-25

**Authors:** Violeta Jevtovic, Asma K. Alshamari, Dejan Milenković, Jasmina Dimitrić Marković, Zoran Marković, Dušan Dimić

**Affiliations:** 1 Department of Chemistry, College of Science, University Ha’il, Ha’il 81451, Saudi Arabia; 2 Department of Science, Institute for Information Technologies, University of Kragujevac, Jovana Cvijića bb, 34000 Kragujevac, Serbia; 3 Faculty of Physical Chemistry, University of Belgrade, Studentski trg 12-16, 11000 Belgrade, Serbia

**Keywords:** pyridoxal thiosemicarbazone, DFT, BSA, cytotoxicity, DNA

## Abstract

Thiosemicarbazones and their transition metal complexes are biologically active compounds and anticancer agents with versatile structural properties. In this contribution, the structural features and stability of four pyridoxal-thiosemicarbazone (PLTSC) complexes with Fe, Co, Ni, and Cu were investigated using the density functional theory and natural bond orbital approach. Special emphasis was placed on the analysis of the donor atom−metal interactions. The geometry of compounds and crystallographic structures were further examined by Hirshfeld surface analysis, and the main intermolecular interactions were outlined. It has been shown that the geometry and the number of PLTSC units in the structure determine the type and contribution of the specific interactions. The binding of all four complexes to bovine and human serum albumin was investigated through spectrofluorometric titration. The dependency of the thermodynamic parameters on the present metal ion and geometry was explained by the possible interactions through molecular docking simulations. The binding of complexes to DNA, as one of the possible ways the compounds could induce cell death, was examined by molecular docking. The cytotoxicity was measured towards HCT116, A375, MCF-7, A2780, and MCF5 cell lines, with Cu-PLTSC being the most active, as it had the highest affinity towards DNA and proteins.

## 1. Introduction

The quest for novel anticancer agents is inspired by the fact that cancer is among the most prominent causes of death worldwide; however, many of the tumors are not treatable by the currently known drugs. This field developed significantly after the introduction of different metals and ligands following the medical use of cisplatin in the 1960s [1,2]. The mechanism of action of cisplatin includes the interference with transcription through binding to the DNA molecules [3,4,5]. However, the use of cisplatin is primarily connected to low selectivity, high toxicity, and acquired resistance. In the last few decades, many transition metal compounds have been examined in vitro and in vivo against cancerous cell lines [6]. 

Thiosemicarbazones (R^1^R^2^C^2^=N^3^-N^2^(H)-C^1^(=S)N^1^R^3^R^4^) represent a group of versatile ligands containing both nitrogen and sulfur donor atoms with a pronounced metal-chelating ability [7,8]. Their preparation includes condensing a ketone or an aldehyde with thiosemicarbazide [9]. Based on the relative orientation of the lone pair of nitrogen respective to the C=S group, the isomers can be *anti* and *syn*, while relative to the C-N bond, there are *cis* (*Z*) and *trans* (*E*) isomers if N and C=S are on the same side or opposite to each other, respectively [10,11]. Their capacity to form metal complexes has intensified their research in medicinal, biological, and pharmaceutical applications [9,12,13]. The binding mode to metals depends on the protonation of different centers, as thiosemicarbazones can be deprotonated/protonated at the –NH_2_ or –SH group [9,14]. The transition metal compounds containing thiosemicarbazone ligands have shown antimicrobial, anti-inflammatory, antitumor [15], antioxidant, and antidiabetic activities [12]. Quang and coworkers investigated the thiosemicarbazone quantum dots for treating Alzheimer’s disease by in silico modeling [16], thus showing the importance of theoretical methods for preparing small-molecular-weight pharmaceuticals. The inhibition of cholinesterase by the *para*-substituted thiosemicarbazones was analyzed in vitro and in silico in the paper by Khan and coworkers [17]. Some of the thiosemicarbazone ligands (marboran or triapine) and their metal complexes have been used in medical practice [18]. Pyridoxal-thiosemicarbazone ligand (PLTSC), an ONS system with an affinity towards various metals, is made as a combination between pyridoxal, a vitamin B_6_ analog, and thiosemicarbazone [19,20]. This ligand was first prepared as a model system for studying the biological interactions of pyridoxal phosphate, a physiologically active form of pyridoxal [20]. A comprehensive review of the complexes with the PLTSC ligand is given in the literature [20].

As a result of their redox inactivity, the Fe, Co, Ni, and Cu complexes have shown good therapeutic and diagnostic properties and versatility when ligand systems are concerned. The ability of these compounds to diffuse through the semi-permeable membrane of cells is responsible for their anticancer activity [7]. Heterocyclic ligands are commonly complexed with these metals as this allows for a high binding affinity to transport proteins (bovine serum albumin (BSA) and humane serum albumin (HSA)), and DNA, selectivity, and considerable cytotoxicity towards certain cancer types [7,21]. Various thiosemicarbazone-transition metal complexes have been exploited in the literature, among which those with Ru, Co, Ni, Pt, Cu, Zn, and Hg deserve special attention [7,12,22]. These complexes are generally mononuclear or binuclear, although complexes of a higher nuclearity are known [7]. Cebotari and coworkers prepared a new class of [Mo_2_O_2_S_2_]-based compounds with these ligands and examined their biological activities [23]. A potent antiproliferation activity against MCF-7 and A549 cancer cell lines was obtained for Ni(II), Cu(II), Pd(II), and Pd(II) complexes with 2-(2,3-dihydroxybenzylidene)-N-ethylhydrazine-1-carbothioamide [24]. Ni complexes with thiosemicarbazones have exhibited a moderate in vitro antimalarial activity that depended on the size of the substituent group [25]. The structures of Fe, Co, Ni, and Cu complexes with PLTSC were previously solved and characterized in the literature [26,27,28,29]. 

The binding of metal complexes to BSA and HSA is an important step in the preliminary investigation of their biological potential [6,30]. These transport proteins have been proven to carry fatty acids, key metal ions, drugs, and toxins [31,32]. HSA consists of 585 amino acid units in a single polypeptide with one tryptophan residue (Trp-214), which is responsible for its fluorescence [33,34,35]. Three predominantly helical domains exist in its structure (I, II, and III), further divided into sub-domains (A and B). The molecular docking study of the DNA binding process is essential in the modern quest for novel pharmaceuticals, as one of the main pathways for the cytotoxic activity of substances includes intercalation or groove binding of compounds to DNA [36]. 

As previously shown, the choice of metal ion determines the stability, geometry, stoichiometry, and presence of different counter ions in complex ions’ internal and external spheres. This article aims to investigate the effect of metal ions (Fe, Co, Ni, and Cu) on the crystal structure and stability of pyridoxal-thiosemicarbazone complexes. Hirshfeld surface analysis was applied to these compounds that differ in the number of ligand molecules and geometry. Density functional theory optimization and natural bond orbital analysis were employed to study the structure and intramolecular interactions governing the stability and reactivity. The binding affinity towards BSA and HSA was investigated by spectrofluorometry, and the thermodynamic parameters of the binding process were determined. The molecular view of the binding process was assessed by the molecular docking simulations towards the mentioned proteins and DNA chains. The cytotoxicity towards selected cell lines (colorectal carcinoma (HCT 116), melanoma (A375), breast cancer (A2780), ovarian cancer (A2780), and healthy lung fibroblasts (MRC5)) was measured and further discussed based on the experimentally and theoretically determined biological interactions. 

## 2. Results

### 2.1. Crystal Structure of Ni-PLTSC Analysis

**Figure 1:**
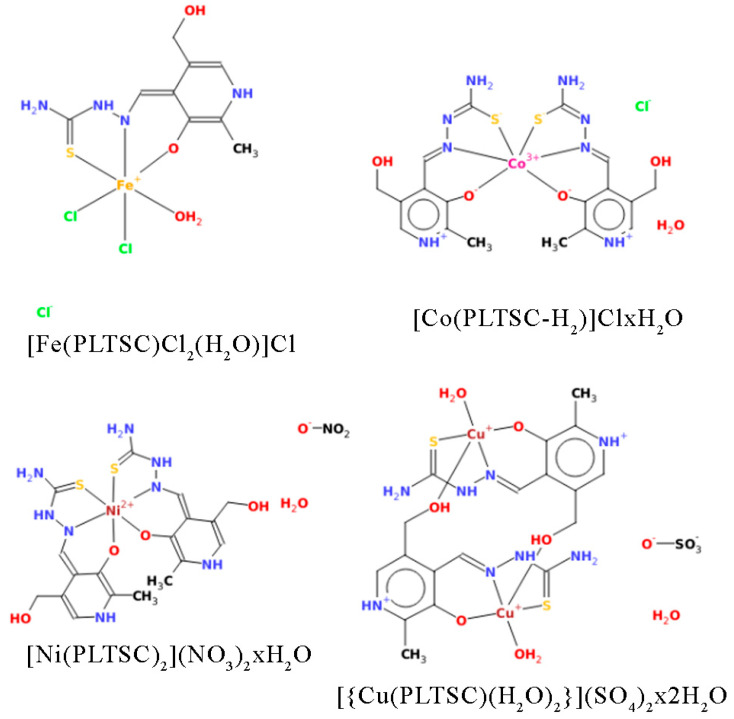
Crystallographic structures of the investigated compounds.

Four complex compounds bearing the same PLTSC ligand were selected to investigate the effect of metal ions on the structural parameters (Figure 1). Fe-PLTSC, Co-PLTSC, and Cu-PLTSC crystal structures were taken from the Cambridge Crystallographic Data Center (CCDC) [28,29,37]. At the same time, the described procedure for synthesizing Ni-PLTSC led to a slightly different structure than the one previously obtained [19]. Chloride salts of iron (III) and cobalt (II), nitrate salt of nickel (II), and copper (II) sulfate were used to synthesize these complexes. The synthesis reaction was carried out in an aqueous solution, as evidenced by crystalline water molecules in the complexes. The iron (III) complex has an octahedral structure with one PLTSC ligand in the coordination sphere, while the remaining three sites are occupied by two chloride ions and a water molecule. Cobalt and nickel complexes are bis-ligand of octahedral geometry, with two PLTSC ligands coordinating with the central ions. In the outer sphere, they have the ions of the starting salts—one chlorine for the cobalt complex and two nitrates for the nickel complex. The only difference between PLTSC co-coordinated ligands for these two bis-ligand complexes is that in the cobalt complex, the ligand is in the monoanionic form, while in the nickel complex, it is in the neutral form. 

The most attractive structure is the copper complex dimer. The unit cells containing two complex compounds are connected by bridges between the central copper atom of one complex and the hydroxyl group of the aromatic pyridine ring of the other. The central copper atom is coordinated with one PLTSC ligand and one water molecule, but its square−pyramidal structure comes from the bridge connecting it to the dimer’s second unit cell, as commonly observed for Cu−thiosemicarbazone compounds [9]. In the outer sphere, a sulfate group neutralizes the central metal charge.

**Figure 2:**
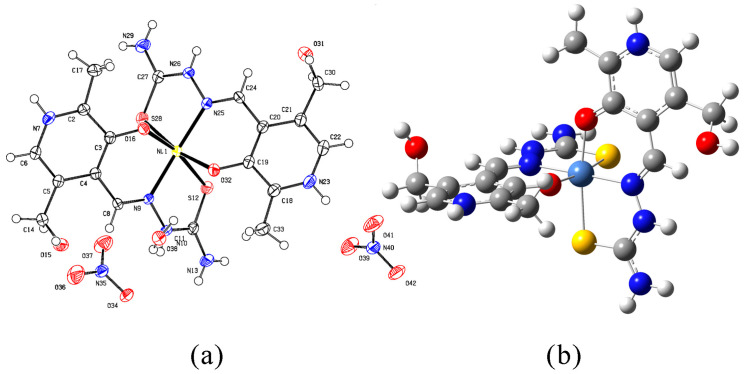
Ni-PLTSC structure (**a**) crystallographic and (**b**) optimized at B3LYP/6-31+G(d,p)(H,C,N,O,S)/LanL2DZ(Ni) level of theory (hydrogen, white; carbon, grey; oxygen, red; nitrogen, blue; sulfur, yellow; nickel, light blue).

The Ni-PLTSC complex was crystallized as a suitable single crystal and underwent an X-ray analysis. It was shown that the Ni complex was chemically identical to the previously published one [19], but now it crystallized with another unit cell parameters so that it was possible to deposit it in the CCDC (deposit number 2262192) as a slightly different structure from the previously synthesized one (Figure 2). The presence of two ONS rings around the central nickel ion and the visible hydrogens on the pyridine nitrogens N7 and N23 (Figure 2a) and the hydrazine nitrogens N10 and N26 (Figure 2b) were evidence of the coordinated ligand in the neutral form. This crystallographic structure was used throughout the article. 

### 2.2. Hirshfeld Surface Analysis

**Figure 3:**
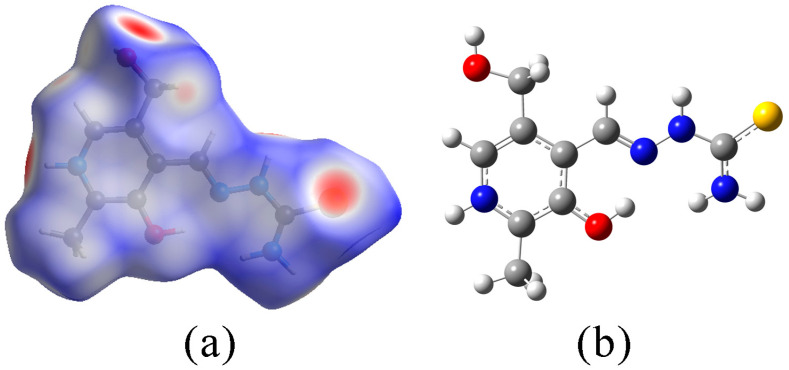
Hirshfeld surface (**a**) and optimized (at B3LYP/6-31+G(d,p) level of theory) structure of PLTSC (**b**) (hydrogen, white; carbon, grey; oxygen, red; nitrogen, blue; sulfur, yellow).

Hirshfeld surface analysis was employed to investigate the effect of the metal ion and coordination on the stabilization interactions within the crystal structure. The crystal structures of the compounds were obtained from the CCDC, as previously mentioned, along with the newly acquired structure of Ni-PLTSC. The fingerprint plots of interactions involving different contact atoms are shown in the Supplementary Material (Figures S1–S5), while the Hirshfeld surface of the PLTSC is presented in Figure 3. 

The crystal structure of the ligand was investigated based on the crystallographic structure from the literature [28]. This analysis was interesting because of the presence of several functional groups responsible for the intermolecular interactions between unit cells. The highest contribution of contacts is denoted as H∙∙∙H (34.1%). These interactions are formed between PLTSC and the surrounding water molecules, as well as between aromatic hydrogen atoms from two PLTSC units. A high contribution was also obtained for H∙∙∙O (13.7%) and H∙∙∙S (15.5%) through interactions between PLTSC and water molecules. A similar percentage of H∙∙∙S was determined for the other thiosemicarbazones [8]. Weak interactions between hydrogen atoms and π electrons of the aromatic ring are included within H∙∙∙C contacts (10.7%). An interaction is formed between the sulfur atom of one PLTSC unit and the hydroxymethyl oxygen of another (S∙∙∙O (1.2%)). The stacking of PLTSC units is possible through the interaction between sulfur atoms and hydrazine nitrogens (S∙∙∙N (2.5%)). Within this analysis, it is essential to observe that the counter ion, chloride, is not included in the interactions, but all of the present functional groups are important for the overall stability. Upon complexation, the hydroxide group is deprotonated, and the hydrazine moiety and sulfur atoms are included in the complex formation, which lowers the contribution of these atoms to the overall stabilization interactions. 

As previously shown, the difference in metal ions leads to different binding of the ligands and other contact points within the crystal structure [20,38,39]. The main contributions to stabilization interactions within the structure of Fe-PLTSC come from the present chloride ions and hydrogen atoms, Cl∙∙∙H (43.6%) and H∙∙∙H (21.4%). This is expected as two chloride ions act as ligands within octahedral geometry, and one chloride ion is a counter-ion in the outer sphere. Chloride ions are also included in other contacts as Cl∙∙∙Cl, Cl∙∙∙S, Cl∙∙∙O, and Cl∙∙∙N, all of which account for less than 1% of contacts. The important contribution comes from contacts including the hydrogen atom, H∙∙∙S (5.6%), H∙∙∙O (8.8%), H∙∙∙C (8.9%), and H∙∙∙N (3.6%). The interactions denoted as H∙∙∙C account for the weak interactions between partially positive hydrogen atom and π electron cloud of aromatic rings [40]. A water molecule that is part of a unit cell also stabilizes the structure through interactions with the surrounding groups. For the complexes containing two ligands, Co-PLTSC and Ni-PLTSC, the main contributions include H∙∙∙H and H∙∙∙O due to the present water molecules within the unit cell. The percentage of H∙∙∙O contacts in the case of Ni-PLTSC is higher than for Co-PLTSC, 37.6 vs. 15.5%, because two water molecules crystallized within the first structure, and one water molecule within the second complex. The amount of H∙∙∙H contacts for Co-PLTSC is 40.4%, while the rest of the contributions, including hydrogen atom are much lower (H∙∙∙S (7.6%), H∙∙∙Cl (11.4%), H∙∙∙N (8.6%), and H∙∙∙C (8.7)). The percentages of contacts, including hydrogen atom in Ni-PLTSC, is very similar (H∙∙∙S (10.4%), H∙∙∙N (5.3%), and H∙∙∙C (9.8)). This shows the importance of co-crystalized solvent molecules for the overall stability of the obtained complexes [41]. The amount of co-crystalized solvent depends strongly on the geometry of complexes and the selected metal ion [6]. Complex Cu-PLTSC is the only one containing metal∙∙∙metal contacts (Cu∙∙∙Cu (0.2%)). The contributions coming from H∙∙∙H and H∙∙∙O are identical (36.5%) due to the abundance of both elements within the structure. A higher percentage of contacts including sulfur atoms, was observed as the counter ion is a sulfate group (H∙∙∙S (5.1%) and S∙∙∙C (3.9%)), while the rest of percentages were comparable to the previous compounds. The DFT methods were further used to explain the differences in structural parameters and intramolecular interactions governing the structures of separate complex ions. 

### 2.3. Structure Optimization and NBO Analysis

The crystallographic structures of the ligands and complexes were optimized at the B3LYP/6-31+G(d,p)(H,C,N,O,S,Cl)/LanL2DZ(metal ions) level of theory. The experimental and theoretical bond lengths and angles are listed in Tables S1, S2 (PLTSC), and S4–S11 (metal complexes). The optimized structures of PLTSC and NI-PLTSC are shown in Figure 2 and Figure 3. The optimized and crystallographic data sets were compared by calculating the mean absolute error (MAE) as a parameter that can be used to verify the applicability of the chosen level of theory [6,42,43]. This parameter presents the average value of the absolute difference between experimental and theoretical bond lengths and angles. In addition, the changes in ligand upon complexation are commented on after comparing the structural parameters of the free and bound PLTSC. 

The structure of PLTSC contains several functional groups that enhance the metal complexation ability [28]. The pyridine ring is substituted by the hydroxyl and hydroxymethyl groups. The aliphatic chain includes a hydrazine group attached to a carbon atom with an amino group and sulfur. The structure of PLTSC is planar due to the extended delocalization from the aromatic ring and electron-donating substituents. The crystal structure bond length and angles, along with the optimized parameters, are given in Tables S1 and S2. The MAE values for bond lengths and angles are 0.02 Å and 1.3°, respectively, proving that the optimized structure represented the experimental one well. When bond lengths are concerned, the highest discrepancy was observed for C-S (0.04 Å) and C-N_amino_ (0.05 Å). These differences are a consequence of the physical state of the sample, as the stabilization interactions within the crystal structure are formed between neighboring units through these groups. Similar results were obtained for the bond angles, as the highest difference between the experimental and theoretical values was calculated for these groups. These findings are in line with the Hirshfeld analysis. As mentioned, electron-donating groups are abundant in the structure of PLTSC, and the second-order perturbation theory was applied to quantify the energies of their stabilization interactions. The most prominent interactions are listed in Table S3. The most numerous interactions can be denoted as π(C-C) → π*(C-C) within the pyridine ring with energies around 12 kJ mol^−1^. The positive resonant effect from nitrogen within the pyridine ring allows for two types of interactions, namely π(N-C) → π*(C-C) and π(C-C) → π*(N-C) with stabilization energies of 76 and 60 kJ mol^−1^. The electron delocalization from the aromatic ring additionally stabilizes the aliphatic chain through the π(C-C)_aromatic_ → π*(C-C) (11 kJ mol^−1^) interaction. The sulfur atom donates electrons to the surrounding groups through strong stabilization interactions, denoted as π(S-C) → π*(N-N) (19 kJ mol^−1^) and π(S-C) → π*(C-N) (17 kJ mol^−1^). The lone pairs of sulfur atoms additionally stabilize groups with stabilization energies of around 50 kJ mol^−1^ (Table S3). Similar results were observed for the amino nitrogen atom and its donation to the S-C bond. The intramolecular hydrogen bond is formed between the hydroxyl group and hydrazine nitrogen atom (LP(N) → σ*(O-H) (93 kJ mol^−1^)). This bond encloses a quasi-six-membered ring within a structure that connects the aromatic ring substituents and aliphatic chain. The deprotonation of this group opens a position for complexation with transition metals, as observed in the studied complexes. A nitrogen atom of the hydrazine group is also important for stabilization through the strongest stabilization interactions observed within the ligand (LP(N) → π*(S-C) (212 kJ mol^−1^) and LP(N) → π*(N-C) (176 kJ mol^−1^)). Upon complexation, the strength of these stabilization interactions is lowered as the donation from the hydroxyl oxygen, hydrazine nitrogen, and sulfur atom are responsible for the formation of compounds. 

Tables S4–S11 show the investigated complexes’ crystallographic and theoretical bond lengths and angles. The chosen theory level’s applicability was verified when the bond lengths and angles were compared. The MAE values for bond lengths were between 0.01 (Co-PLTSC) and 0.04 Å (Fe-PLTSC and Cu-PLTSC), which is of the order of the experimentally determined uncertainties [19]. The MAE values for bond angles were somewhat higher than the experimental uncertainties, between 1.2 (Co-PLTSC) and 2.9° (Cu-PLTSC). Careful inspection of the bond angles explained these values. The bond angles around the metal ions showed the highest difference between the experimental and theoretical values. In the case of Fe-PLTSC, the angles formed between chloride, iron, and oxygen changed from 104.2 to 89.1°. Similar results were obtained for Cu-PLTSC, in which angle S-Cu-O3 changed from 150.4 to 172.2°. This is a consequence of the optimization performed on the isolated complex ions in a vacuum without additional ions and water molecules. As previously described, the crystallographic structures were obtained in the solid phase with abundant intermolecular interactions. All of the investigated complex compounds have a pseudo-octahedral geometry that is restored upon optimization. A similar geometry has been found in other thiosemicarbazone complexes [44]. Complexes containing Ni and Co surrounded by two PLTSC units show the lowest difference between experimental and optimized bond lengths, as the solvent molecules and surrounding compounds do not significantly influence the geometry. This type of analysis led to optimized structures that were further used for molecular docking. 

It is important to investigate what happened with the structural parameters of PLTSC upon complexation. For this type of complex to be formed, rotation around the C−N bond occurred, leaving the sulfur atom on the same side as the hydroxyl group of the aliphatic chain and forming the tridentate ligand. The most noticeable differences in bond lengths were determined for the groups included in complex ion formation. Once the hydroxyl group of the aromatic ring was deprotonated, the C−O bond length decreased from 1.33 Å (PLTSC) to 1.26 Å (Fe-PLTSC). The presence of metal ion changed the bond lengths of the aliphatic chain to allow for the formation of three bonds; for example, the N-N bond length increased from 1.33 (PLTSC) to 1.38 (Fe-PLTSC), and the C−N bond length decreased from 1.40 (PLTSC) to 1.35 Å (Fe-PLTSC). Much lower differences in these changes were obtained in the case of Co and Ni, which is a consequence of the ionic radii of the investigated compounds. These findings give a quantitative reason for the changes in ligand structure upon complexation, which can be important for the interactions with the biomolecules [21].

The stabilization interactions between metal ions and atoms donating free electron pairs were quantified by the second-order perturbation theory. The most significant structural change between free and bound ligands is the absence of a hydrogen atom on the hydroxyl group and the absence of a hydrogen bond that stabilizes the structure between the aromatic ring and the aliphatic chain. The selected stabilization interactions are listed in Table S12. The structure of Fe-PLTSC is stabilized by the interaction between oxygen, nitrogen, and sulfur atoms and Fe through LP(O) → σ*(Fe-Cl) (51 kJ mol^−1^), π(C-S) → LP*(Fe) (18 kJ mol^−1^), and π(C-N) → LP*(Fe) (22 kJ mol^−1^). A strong interaction was observed between a water molecule and Fe ion (LP(O_water_) → LP*(Fe)) with a stabilization energy of 355 kJ mol^−1^, proving the importance of water molecules for the overall stabilization of the structure. In an aqueous solution, these bonds are expected to be distorted, leading to decreased stability of the compounds. In the case of complex compounds with two PLTSC units, additional interactions were obtained between two ligand molecules. The stabilization interactions also include mentioned atoms, although they are much stronger for Ni-PLTSC. The NBO analysis confirmed that the structure of Cu-PLTSC consists of two units connected by a bridging interaction between the hydroxymethyl oxygen atom of one unit and Cu ion of the second (LP(O) → LP(Cu2)), with a stabilization energy of 100 kJ mol^−1^. The oxygen, nitrogen, and sulfur atoms of PLTSC stabilize Cu ion through the same type of interactions as previously described, with stabilization energies between 43 (LP(N) → LP*(Cu)) and 107 kJ mol^−1^ (LP(O) → LP*(Cu)). In all of the structures, the interactions between the oxygen atom attached to an aromatic group and metal ion are the strongest, proving the importance of PLTSC deprotonation for stability. The interactions within the PLTSC ligand are similar to the ligand in free form. 

### 2.4. Spectrofluorometric Investigation of the Binding to BSA and HSA

**Figure 4:**
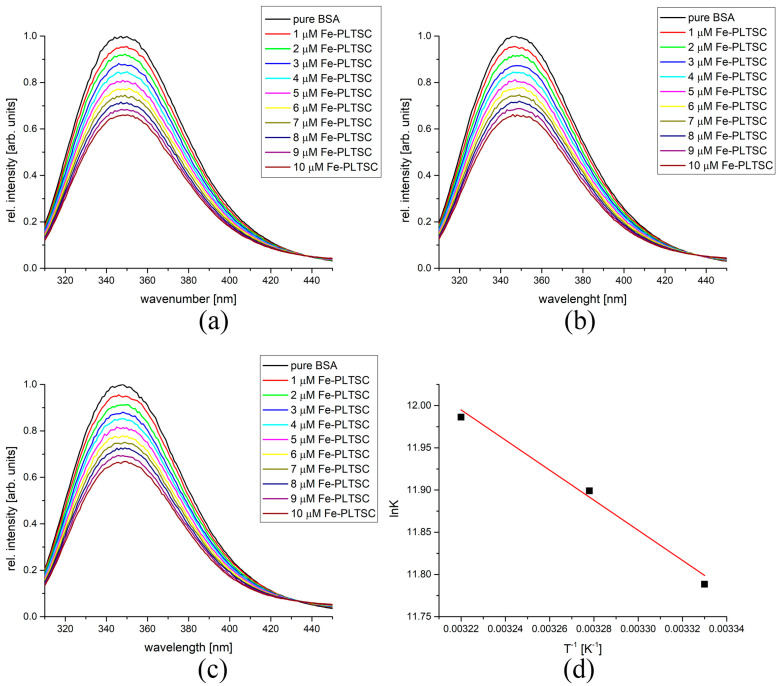
The fluorescence spectra of BSA after the addition of various amounts of Fe-PLTSC at (**a**) 27 °C, (**b**) 32 °C, (**c**) 37 °C, and (**d**) Van’t Hoff diagram for the binding process of Fe-PLTSC and BSA.

The binding of the investigated complexes to BSA and HSA, as important transport proteins, was analyzed by spectrofluorometric titration. BSA and HSA were irradiated by an excitation wavelength of 295 and 280 nm, respectively, to activate the intrinsic fluorescence of tryptophan and tyrosine residues at the active positions. The change in the secondary structure of proteins led to a change in the chemical environment in the active pocket, and the fluorescence intensity decreased upon binding. As an example of the binding experiments, the fluorescence spectra of BSA before and after the addition of Fe-PLTSC are shown in Figure 4. As can be seen, the fluorescence intensity decreased in a concentration-dependent manner. The binding constants were determined from the double-log Stern−Volmer plots as a dependency of intensity vs. concentration of the complex. The number of complexes bound to BSA could be determined from this analysis. The binding constants at three temperatures, along with the number of molecules bound to BSA and thermodynamic parameters for four complexes, are given in Table 1.

Upon the addition of the investigated compounds, the fluorescence intensity decreased. In the case of Fe-PLTS, the plots were linear for the dependency between fluorescence intensity vs. concentration, with a high correlation coefficient (between 0.993 and 0.997). The number of binding sites was almost equal to 1, proving that the newly formed structures could be denoted as Fe-PLTS-BSA. The values of the binding constant were of the order of 10^5^ M^−1^, which is comparable to pyridine-based Fe complexes [45]. The positive value of binding enthalpy change (15.5 kJ mol^−1^) suggests that the fluorescence quenching occurred through a static mechanism, as shown for other Schiff base Fe compounds [46]. The positive values of enthalpy and entropy change for Fe-PLTSC, Co-PLTSC, and Cu-PLTSC complexes indicate entropy-driven binding of molecules in which entropy is a measure of the dynamics of the overall system reflecting the changes in the translational and rotational degrees of freedom of the interacting molecules and eventually causing changes in their movement within the formed complexes. As the absolute values of the free energy change for these complexes increase with increasing temperature, a thermodynamically more favorable reaction at higher temperatures is indicated. For the investigated temperature interval, the binding process between Fe-PLTSC and BSA was spontaneous, between −29.4 and −30.9 kJ mol^−1^, similar to those obtained for the iron complex with imidazole derivatives [47].

Once the number of ligand molecules increased, as in Co-PLTSC, the number of binding sites remained the same (Table 1), but the values of binding constants increased. The binding enthalpy and entropy changes were still positive, 70.3 and 33.7 kJ mol^−1^, respectively. There was a decrease in the entropy change, but an increase in enthalpy change. The spontaneity of the process resembled that of a Co(II)-1,10-Phenanthroline complex, as determined by Zhao and coworkers [48]. These changes signify the importance of the newly formed interactions between the second ligand molecule and the surrounding amino acids. The following section investigates the binding mode through molecular docking simulations. A complex containing Ni also spontaneously bound BSA with a more negative ΔG_b_ value (from −41.7 to −40.4 kJ mol^−1^ in the examined temperature range) compared with the other two complexes. This is the only process with a negative value for both enthalpy and entropy changes, indicating that van der Waals and hydrogen bond interactions were dominant between Ni-PLTSC and BSA. These weak interactions were the consequence of the protonation state of the ligand, as previously mentioned. PLTSC is monoanionic within Co-PLTSC while neutral within Ni-PLTSC, which limits the possible interaction types with the surrounding amino acids. The negative values of change in enthalpy and entropy of binding to BSA were also obtained for the Ni(II)-Schiff base complex in the paper by Ray and coworkers [49], which led to the conclusion that hydrogen bonding and van der Walls interactions were dominant for the binding process.

The most spontaneous binding process was observed for the Cu-PLTSC complex, between −38.9 and −42.5 kJ mol^−1^, in the given temperature range. This result is expected as the number of ligands and metal ions exceeds the previously analyzed compounds. The number of binding sites remained 1, with the change in enthalpy being 67.4 kJ mol^−1^ and the change in entropy being 355 J mol^−1^ K^−1^. The obtained values show that the interaction strength was comparable to the other three compounds, but the binding process induced a significant decrease in systems order, thus being positive. This high ΔS value signifies the decrease in the system’s order as the interaction strength and number between protein and complex were much lower than in the crystal structure of Cu-PLTSC, as explained in the previous sections. ΔG_b_ values between −37.4 and −40.0 kJ mol^−1^ were obtained for binding the stable Schiff base-copper complex to BSA at a temperature range between 290 and 310 K, which coincides well with the present results [50].

The competitive experiments with warfarin were further performed to determine the active position these compounds bind to. This marker was selected as it was shown to have a greater impact on the binding processes than the other site-specific markers [33]. Table S13 lists the binding constants and the number of binding sites for the experiments, including warfarin. The binding constants of Fe-PLTSC and Ni-PLTSC were almost equal to those obtained in the experiments without warfarin, indicating that the binding process was not affected by the presence of this marker. This led to the conclusion that the complexes bind to BSA’s subdomain IIIA. The reduction percentages of the binding constants in the case of Co-PLTSC (5.25 × 10^4^ M^−1^) and Cu-PLTSC (3.39 × 10^6^ M^−1^) were 10 and 23%, respectively. This result shows that these two complexes bind to subdomains IIA and IIB [9,10]. It can be assumed that the active pocket size determines a specific site’s preference. 

**Figure 5:**
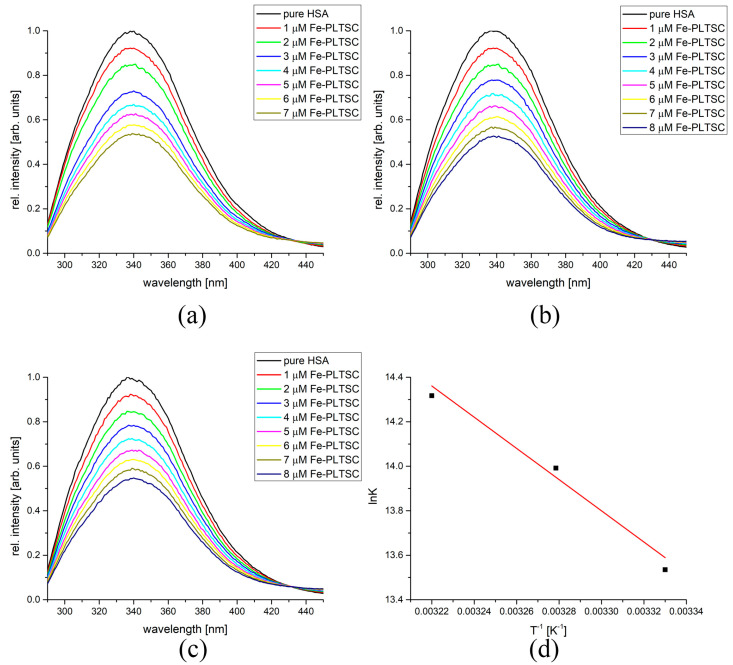
The fluorescence spectra of HSA after the addition of various amounts of Fe-PLTSC at (**a**) 27 °C, (**b**) 32 °C, (**c**) 37 °C, and (**d**) Van’t Hoff diagram for the binding process of Fe-PLTSC and HSA.

The binding of the investigated compounds to HSA followed a similar behavior. Figure 5 shows fluorescence curves and Van’t Hoff plot for the binding of Fe-PLTSC to HSA, while Table 2 lists the most important thermodynamic parameters. As with Fe-PLTSC-, Co-PLTSC-, and Cu-PLTSC-BSA complexes, the absolute values of the free energy change increased with the increasing temperature, indicating the reaction as thermodynamically more favorable at higher temperatures. The values of the binding constants in the case of Fe-PLTSC binding to HSA were higher than for the same process involving BSA, showing that the chemical environment of the florescent amino acids was more influenced by the presence of metal ions. The positive change in the enthalpy and entropy of binding (58.3 kJ mol^−1^ and 307 J mol^−1^ K^−1^) again points out that the weak interactions were the most important for the binding. The values of ΔG_b_ were somewhat higher, indicating higher spontaneity of the binding process. The binding of Co-PLTSC to HSA showed similar values for the change in the Gibbs energy of binding. As the only example of this behavior type, the negative slope within the analyzed temperature range for Ni-PLTSC was the same as for the previously investigated protein. As expected, the highest binding affinity was determined for Cu-PLTSC (between −42.6 and −46.3 kJ mol^−1^) due to the complex structure. The differences in the binding affinities are explained in the next section using molecular docking methods.

### 2.5. Molecular Docking Investigation of the Binding Process between Investigated Complexes and BSA, HSA, and DNA

**Figure 6:**
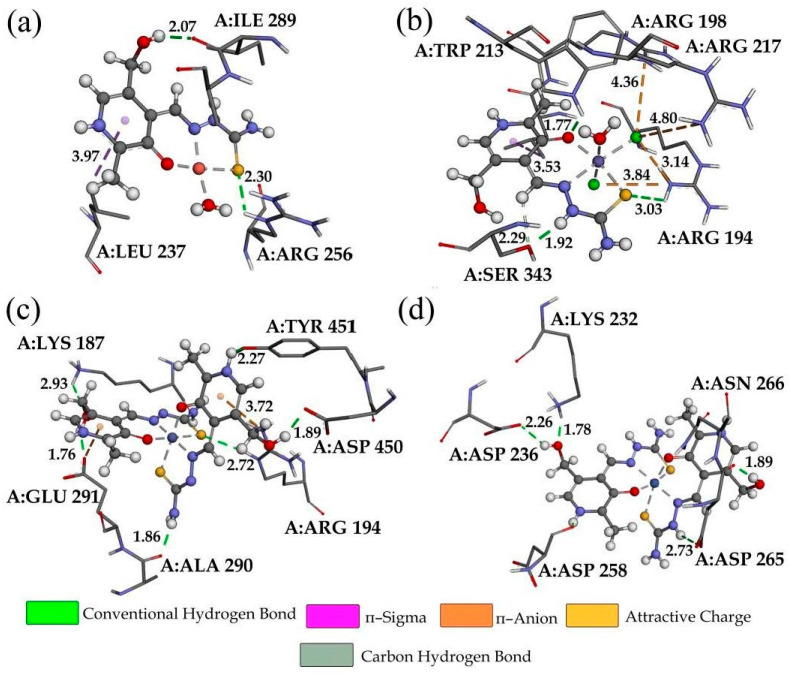
The most stable conformations of investigated complexes in the structure of BSA (active pocket IIA) (**a**) Cu-PLTSC, (**b**) Fe-PLTSC, (**c**) Co-PLTSC, and (**d**) Ni-PLTSC.

The binding of the investigated compounds to BSA at the molecular level was analyzed through molecular docking. It is well established that the complex compounds bind through hydrophobic cavities in subdomains IIA and IIIA [46]. The experimental BSA binding results were accompanied by the modeling of different adducts between the investigated compounds and BSA. The most stable conformations of adducts are examined in this section. Both active pockets (IIA and IIIA) were included in this study, and the main findings are shoown in Table 3 with the free binding energy and its contributions. The most important interactions are depicted in Figure 6 and Figure S6. The calculated binding energies range between −18 and −24.6 kJ mol^−1^ for all of the investigated systems. Compound Fe-PLTSC binds to IIA with a binding energy of −24.4 kJ mol^−1^ and IIIA with a binding energy of −20.3 kJ mol^−1^. These results show that the binding of Fe-PLTSC is spontaneous at both positions. The discrepancy between these values and the experimental ΔG value is noticeable. The main reason is that BSA’s crystallographic structure significantly differs from the one in the aqueous solution [46], as explained in the literature [50,51]. Furthermore, the complex structure of Fe-PLTSC included in the docking study contained chloride ions that are probably not bound to the central metal ion once dissolved. A close-up of the possible interactions (Figure 6) revealed that Fe-PLTSC is surrounded by TRP213, ARG194, ARG193, ARG217, and SER343 amino acid residues. It is important to mention that TRP213 is a fluorescent amino acid partially responsible for BSA fluorescence, showing that the molecular docking results coincide well with the experimental ones. Chloride anions form π-anion interactions with ARG194 and ARG198 residues, which cannot be expected in an aqueous solution. The strongest interactions are the conventional hydrogen bonds formed between the PLTSC and SER343/ARG194 polar groups. Other interactions include π → σ and carbon−hydrogen bonds. The surrounding amino acids within the active pocket IIIA include LEU189, GLU186, THR190, and SER428 (Figure S6). Again, the interactions with anions were observed. The difference in the binding energies within two pockets lies in the number of hydrogen bonds that are formed, as can be seen in Table 2 when $\Delta G_{\mathrm{vdw}+\mathrm{hbond}+\mathrm{desolv}}$ contributions are compared (−28.6 kJ mol^−1^ for IIA and −23.7 kJ mol^−1^ for IIIA). 

Compounds containing two PLTSC ligands have lower free binding energies for both cavities. In the case of Ni-PLTSC, these values were −20.5 (IIA) and −18.0 kJ mol^−1^ (IIIA), while for Co-PLTSC they were −19.9 (IIA) and −17.0 kJ mol^−1^ (IIIA) (Table 3). Both compounds were bound to similar amino acids as Fe-PLTSC (Figure 6 and Figure S6). As previously explained, the lack of negatively charged ions in the structures of these complexes limits the types of interactions formed to conventional hydrogen bonds, π → σ, and carbon−hydrogen bonds, which is reflected in the highest contribution of $\Delta G_{\mathrm{vdw}+\mathrm{hbond}+\mathrm{desolv}}$ in the total value. The polar groups that were positioned outwards from the complex compound center formed hydrogen bonds; for example, an amino group with ALA290 (Co-PLTSC) or hydroxyl hydrogen with ASP-258 (Figure 6) [33]. These two compounds had higher torsional energies than the others, as ligands are not as tightly bound as within Fe-PLTSC (Table 3). 

The highest binding free energies were calculated for Cu-PLTSC, which coincides well with the experimental data (Table 3). If Fe-PLTSC is excluded due to the presence of chloride ions in the structure, the values of binding free energy follow the experimental order (Cu-PLTSC > Ni-PLTSC > Co-PLTSC) showing that molecular docking can be used for the prediction of affinity order with all of the limitations previously explained. Cu-PLTSC is surrounded by ILE289, LEU237, and ARG256 by forming hydrogen bonds between hydroxymethyl oxygen and an amino group of ILE289, and between the sulfur atom and ARG256 (Figure 6).

**Figure 7:**
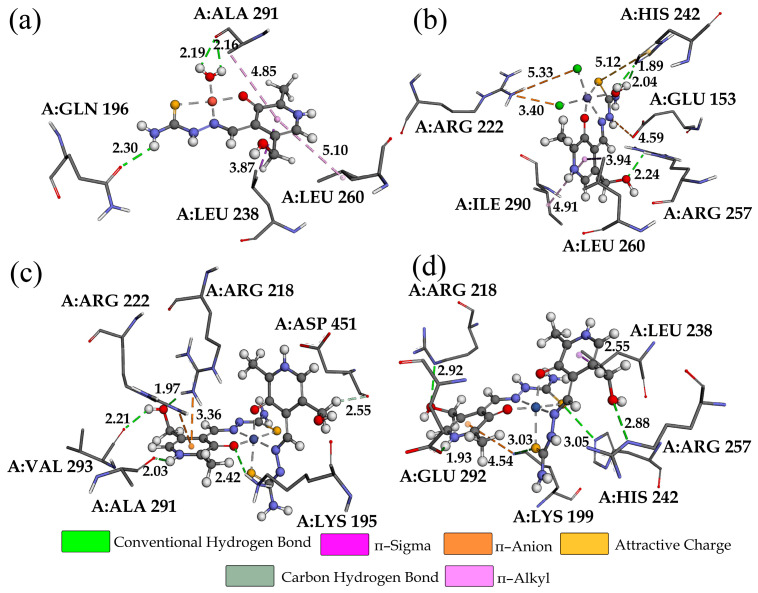
The most stable conformations of the investigated complexes in the structure of HSA (active pocket IIA): (**a**) Cu-PLTSC, (**b**) Fe-PLTSC, (**c**) Co-PLTSC, and (**d**) Ni-PLTSC.

The binding of the investigated compounds was investigated towards HSA to elucidate the location of the binding site [33]. The free energies of binding are presented in Table S14 with the main contributions, while Figure 7 and Table S7 show the most important interactions. Again, these values are in the narrow range between −24.0 (Co-PLTSC) and −19.1 kJ mol^−1^ (Ni-PLTSC) for domain IIA and between −23.5 (Cu-PLTSC) and −13.1 kJ mol^−1^ (Ni-PLTSC) for domain IIIA. When the overall binding affinity is concerned, complex Cu-PLTSC most was most spontaneously bound to HSA (−23.4 kJ mol^−1^ for domain IIA and −23.5 kJ mol^−1^), which is consistent with the experimental findings. The theoretical free energy of binding was lower than the experimental (−46.3 kJ mol^−1^) value because the conformation of HSA in solution was significantly different from the crystallographic one, and the solvent effect additionally influenced the binding process. When the binding mode of Cu-PLTSC was concerned, hydrogen bonds in IIA were formed between water molecules of a complex with ALA291 (2.19 and 2.16 Å) and between the amino group and GLN196 (2.30 Å). Other interactions include π-alkyl and π-σ with LEU260 and LEU238. The binding affinity of Fe-PLTSC towards HSA was overestimated due to the presence of chloride ions that were part of the crystal structure, which formed attractive charge interactions with ARG222 and HIS242. These interactions were characterized by longer interatomic distances (5.33 and 2.40 Å). Similar spontaneity of the process was observed for the binding of Fe-PLTSC to both active domains. The same active position and similar interactions were observed in the crystal structure of HSA bound Fe complex with N,N-bis(2-pyridinylmethyl)-2,20-bipyridine-6-methanamine) [52]. The analyzed confirmation of Co-PLTSC was positioned so that an abundance of interactions were formed with the surrounding amino acids; for example, conventional hydrogen bonds with VAL293, ALA291, and LYS195 through polar groups of PLTSC. The aromatic rings of ligands were included in the attractive charge, π-alkyl, and π-σ interactions. Similar results were observed for Ni-PLTSC, proving the importance of the geometry of compounds for the binding affinity. The lower binding affinity in the subdomain IIIA resulted from the lower number of conventional hydrogen bonds. The results implicate that all complexes interacted with th TYR411 amino acid, a weak fluorescence emitter. More importantly, all of the complexes were found in the vicinity of TRP214, which was responsible for the most fluorescent emission, and any change in its surrounding affected the intensity, as observed experimentally [33,53].

**Figure 8:**
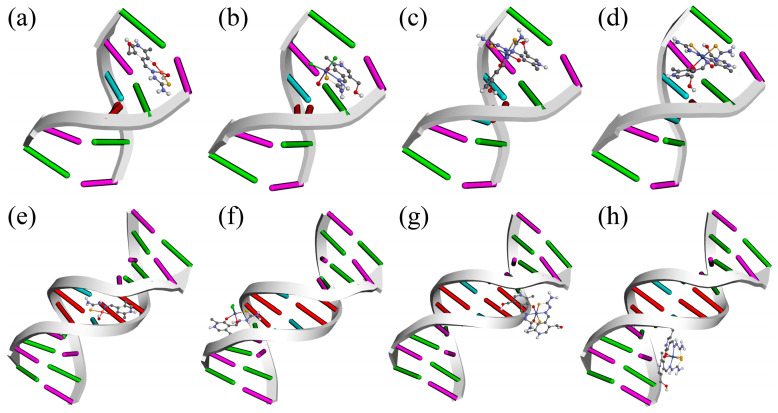
The most stable conformations of investigated compounds in six-base-pair DNA (upper row): (**a**) Cu-PLTSC, (**b**) Fe-PLTSC, (**c**) Co-PLTSC, and (**d**) Ni-PLTSC. Dodecamer DNA (lower row): (**e**) Cu-PLTSC, (**f**) Fe-PLTSC, (**g**) Co-PLTSC, and (**h**) Ni-PLTSC.

The interactions between the complexes and DNA were investigated in a preliminary study of their potential biological activity and cytotoxicity. Many of the anticancer regiments include the DNA-binding process [36]. For this molecular docking study, two systems were taken, one with six base pairs (upper row, Figure 8) and the other with ten base pairs (lower row, Figure 8). The complexes could adopt four positions in each DNA chain, entering interactions of different strengths. Regarding the six-base DNA, the compounds fit into the structure with binding affinities between −23.3 (Co-PLTSC) and −23.9 kJ mol^−1^ (Cu-PLTSC) (Table S15). The interactions with dodecamer were much more diverse, as seen in Figure 8. These interactions were somewhat stronger, between −26.0 (Co-PLTSC) and −31.9 kJ mol^−1^ (Cu-PLTSC) (Table S15). These interaction energies were similar to those calculated for the polypyridyl Fe(II) and Fe(III) complexes that were experimentally confirmed [54]. It should be noted that the positive overall charge of compounds enhanced the interactions with DNA as the DNA possesses an anionic phosphate backbone [55]. 

**Figure 9:**
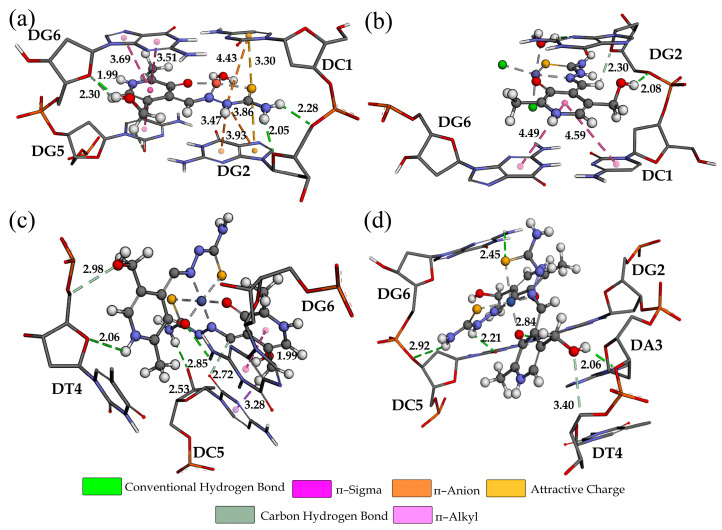
The interactions formed between investigated complexes and six-base-pair DNA for the most stable conformers: (**a**) Cu-PLTSC, (**b**) Fe-PLTSC, (**c**) Co-PLTSC, and (**d**) Ni-PLTSC.

The structure of DNA with six base pairs was investigated first. As seen in Figure 9, four compounds adopted different positions depending on the geometry of the complexes. The most stable interactions were formed between Cu-PLTSC and DNA (−29.3 kJ mol^−1^). The distribution of ligands around the central metal ion led to the planarity of compounds that could be intercalated between two base pairs. Hydrogen bonds are formed within this conformer with DG6, DC1, and DG2. The attractive charge interaction was observed between DC1 and the sulfur atom. The binding affinity in the case of the other three compounds was almost equal (−25.9 (Ni-PLTSC), −24.0 (Fe-PLTSC), and −23.3 kJ mol^−1^ (Co-PLTSC)) and was dependent mainly on the number of hydrogen bonds (Table S15). The intercalation of these compounds into DNK was much less spontaneous due to the size of the molecule [56]. The aromatic ring of Fe-PLTSC interacted with DG6 and DC1 through weak π-alkyl and π-σ interactions, while the hydrogen bond formed between hydroxymethyl hydrogen and DG2 (2.08 Å). The Co and Ni complexes formed interactions through polar groups with DG6, DC5, and DT4 residues. These results prove that the biological properties depend on the geometry, charge, size, and nature of the metal ions [55]. 

The best docking pose for the investigated compounds in the case of dodecamer DNA is shown in Figure 8 and is examined in more detail in Figure S8. It is indicated that different complexes with PLTSC ligands were bound to various positions along the chain, thus proving the importance of geometry for the most stable position. Compound Cu-PLTSC was bound to the curved contour of the targeted DNA in the minor groove, similar to what Shamsi and coworkers observed [57]. The intercalation is traditionally associated with the planar molecules with fused bi/tricyclic structures, which covers the structural features of Cu-PLTSC. Intercalators have been investigated for their antitumor, antimalarial, and antifungal properties [36]. The binding energy between Cu-PLTSC and DNA was −31.9 kJ mol^−1^, with the strong intercalating effect determined by the hydrogen bond formation with DT8, DT9, and DT18 [56] (Table S15). The interatomic distances of hydrogen bond interactions were between 2.14 and 2.52 Å. The carbon−hydrogen bonds were formed between DT9 and Cu-PLTSC as much weaker bonds. Compound Fe-PLTSC was partially surrounded by the base pairs with chloride ions positioned contrariwise, with a binding energy of −26.9 kJ mol^−1^. The interactions of this complex were formed between hydroxymethyl oxygen and DC23 (1.67 Å), amino group and DT20 (2.12 Å), water molecules and DG22 (2.09), and hydrazine groups hydrogen and DG22 (2.31 Å) (Figure S8). Because of the possibility of the weaker bond formation with the surrounding groups, the remaining two compounds were docked outside the DNA molecule. The binding energies reflect the position that included interactions with only one part of ligand (−26.0 kJ mol^−1^ (Co-PLTSC) and −26.5 kJ mol^−1^ (Ni-PLTSC)). This so-called grove binding is characteristic of the crescent-shaped molecules that can be easily twisted [36], such as the investigated complexes of Co and Ni. These results show that the geometry of compounds is much more important for the intercalating ability than the functional groups present. 

### 2.6. Cytotoxicity Studies

The cytotoxicity of the investigated complexes was examined towards A375 melanoma, MCF-7 breast cancer, HCT116 colorectal carcinoma, A2780 ovarian cancer, and MRC5 lung fibroblasts) using two methods, MTT and CV. These cell lines were selected as the common cancer types. Table 4 presents IC_50_ values with standard deviation, while the dose−response curves are given in Figures S9–S14. 

The results presented in Table 4 show that, in the investigated concentration range, Fe-PLTSC, Co-PLTSC, and Ni-PLTSC were not active towards the analyzed cell lines, except for the low toxicity of Fe-PLTSC towards A2780 (86.3 ± 13.1 (MTT) and 93.5 ± 9.2 (CV)). On the other hand, Cu-PLTSC was active in all four cancer cell lines. Figures S9–S14 prove that the viability changed in a concentration-dependent manner. The results obtained by the MTT and CV tests were similar, proving the measurements were done well. The highest activity was measured towards the A2780 cell line with IC_50_ values of 1.8 (MTT) and 1.7 μM (CV). For the other cell lines, the IC_50_ value was between 17.5 (HCT 116) and 74.6 μM (A375). It is important to point out that the activity of Cu-PLTSC was higher than that of cisplatin (33.6 ± 4.8 μM) as measured by the MTT test towards MCF-7 [58]. These results coincide well with the preliminary results on the interactions with transport proteins and DNA. The geometry of complex and conformation of ligands around metal ions play a crucial role in the measured cytotoxicity, and the results of the in silico study are the first step in the forthcoming biological studies on Cu-PLTSC. The activity of Cu-PLTSC against MRC5 allowed for the examination of the selectivity. Based on these results, it can be concluded that Cu-PLTSC is more active toward ovarian cancer cells than healthy lung fibroblasts, which paves the way for the investigation into the types of cancer this compound should be investigated on.

## 3. Materials and Methods

### 3.1. Chemicals

All of the chemicals used throughout experiments (Ni(NO_3_)_2_, CoCl_2_∙6H_2_O, CuSO_4_∙5H_2_O, FeCl_3_∙6H_2_O, pyridoxal, thiosemicarbazide, Bovine Serum Albumin (BSA)), and the solvents (ethanol, DMSO) were obtained from Merck as *p*.*a*. purity chemicals. The ultra-pure water was used for the spectrofluorometric measurements (Milli-Q^®^ EQ 7000 ultrapure water system). Fetal bovine serum (FBS) and culture mediums RPMI-1640 and Dulbecco’s Modified Eagle Medium (DMEM) were purchased from Capricorn Scientific GmbH (Hessen, Germany). Penicillin Streptomycin solution was purchased from Biological Industries (Cromwell, CT, USA). Trypsin, phosphate-buffered saline (PBS), and dimethyl sulfoxide (DMSO) were purchased from Sigma (St. Louis, MO, USA). 3-(4,5 dimethythiazol-2-yl)-2,5-diphenyltetrazolium bromide (MTT) was purchased from AppliChem (Maryland Heights, MO, USA). Paraformaldehyde (PFA) was purchased from Serva (Heidelberg, Germany). Crystal violet (CV) was purchased from Biowest (Riverside, MO, USA).

### 3.2. Synthesis of Investigated Compounds

The mixture of 0.20 g (0.7 mmol) of Ni(NO_3_)_2_ and 0.15 g (0.5 mmol) of PLTSC × 3H_2_O was poured over with 5 cm^3^ H_2_O and dissolved by heating, similar to the previously described procedure [19]. After 24 h, the obtained brown crystals were filtered and washed with H_2_O and ethanol. Yield: 0.20 g (58%). For the other three compounds, the procedure described in the literature was applied [19,28,29,37]. The compounds were recrystallized and used as obtained.

### 3.3. X-ray Analysis

A single crystal of Ni-PLTSC with dimensions of 0.12 × 0.09 × 0.17 mm was separated and analyzed (Table 5). The X-ray investigation was done on a single crystal mounted on glass fiber and examined at 173 K. The instrument used was a Bruker D8 Venture APEX diffractometer equipped with Photon 100 area detector using graphite-monochromator Mo-Kα radiation [λ = 1.54184 Å]. The absorption corrections were done with the SCALE3 ABSPACK algorithm implemented in the CrysAlisPro software (Rigaku, Cedar Park, TX, USA) [59]. The locations of hydrogen atoms were obtained from a difference map. These atoms were initially positioned geometrically, while the hydrogen atoms for the coordinated water molecules were found through the difference map. The positions were then refined in separate cycles. Hydrogen positions were checked for feasibility by examination of the hydrogen-bonding network. The crystallographic data were deposited in the Cambridge Crystallographic Data Centre (CCDC, Cambridge, UK) under CCDC number 2262192. Crystal data collection and structure refinement are given in the following table.

### 3.4. Hirshfeld Surface Analysis

Hirshfeld surface analysis is a method for theoretically investigating the obtained crystal structures. It proved useful for determining and quantifying the intermolecular interactions governing the stability of the structure. This analysis was performed in the CrystalExplorer program package [60]. Hirshfeld surface analysis represents a graph connecting two distances; the first one is the distance between the two nearest nuclei (de), while the second is the distance from nuclei to the external surface (di) [61,62,63]. The distances are normalized and colored depending on the van der Waals radii separation between atoms. The red, white, and blue values represent these values if the distance is shorter, equal, or longer than the van der Waals radii. The normalized distances in this paper are given between −0.4939 a.u. (red) and 1.1572 a.u. (blue). Fingerprint plots give the relative positions and percentages of the specific combination of atoms and are important for understanding the stabilization interactions.

### 3.5. Theoretical Calculations

The structures of the investigated complexes were optimized in the Gaussian Program Package (Gaussian 09, Revision C 01) [64]. The newly obtained Ni complex crystal structure and other complexes’ available crystal structures were used for the optimization. The Global Hybrid Generalized Gradient Approximation (GGA) functional B3LYP [65] was applied for the analysis, along with the 6-31+G(d,p) [66] basis set for H, C, N, O, S, and Cl atoms and LanL2DZ basis set for Fe, Co, Ni, and Cu ions [67,68]. The same level of theory was previously successfully applied to the structural analysis and spectral assignations of similar compounds [42,43]. The optimizations were performed without any geometrical constraints, and the minima on the energy surface were found, as proven by the absence of imaginary frequencies. Several spin states for the included metals were chosen, and only the most stable structures were analyzed in this contribution. The vibrational analysis employed the visualization in the GaussView 6 program (Gaussian Inc., Wallingford, CT, USA) [69]. The intramolecular interactions governing stability were investigated using Natural Bond Orbital Analysis [70,71]. 

### 3.6. Spectrofluorometric Measurements

The binding affinities of complexes towards bovine serum albumin (BSA) and human serum albumin (HSA) were analyzed using the spectrofluorometric measurements on the Cary Eclipse MY2048CH03 instrument. The scan rate was set to 600 nm min^−1,^ with both slits being 5 nm. The excitation wavelength for BSA was set to 295 nm and for HSA to 280 nm. These wavelengths are characteristic of the tryptophan residues in the active pocket of proteins. The spectra were recorded from 310 to 500 nm. The concentration of both proteins was held constant at 5 × 10^−6^ M, and prepared in 1 M phosphate buffer saline of pH 7.4. The concentration of the investigated compounds ranged between 1 and 10 × 10^−6^ M. The data were analyzed according to the double log Stern−Volmer quenching (Equation (1)):

(1) $$\log\left(\frac{I_{0}-I}{I}\right)=logK_{b}+nlog\left[Q\right]$$

where *I*_0_ and *I* are the fluorescent emission intensities of BSA without and with added metal complexes, respectively; *K_b_* is the binding constant; *n* is the number of binding places; and [*Q*] is the concentration of quenchers (in this case metal complexes).

The measurements were repeated at three temperatures (27, 32, and 37 °C) to mimic the body temperature and to allow for the calculation of the thermodynamic parameters of binding using the vant’t Hoff equation (Equation (2)):

(2) $${\mathrm{lnK}}_{b}=-\frac{\Delta H_{b}}{RT}+\frac{\Delta S_{b}}{R}$$

where Δ*H_b_*, Δ*S_b_*, and Δ*G_b_* represent the change in the enthalpy, entropy, and free energy of the binding process, respectively. 

The competitive measurements were performed in the presence of warfarin, a site-specific marker for BSA subdomain IIA [33]. The equimolar concentrations of BSA and warfarin were prepared (5 × 10^−6^ M), and after 30 min of incubation, the titrations using the solutions of the investigated complexes were conducted [30]. The final concentrations of the added complexes were the same as for the previous experiments.

### 3.7. Molecular Docking

The molecular docking analysis complemented the spectrofluorometric measurements in order to understand the binding of the investigated complexes towards BSA and HSA and the additional examination of the DNA binding affinities. The Autodock 4.2 software [72] with the Lamarckian Genetic Algorithm (LGA) was employed to determine the binding affinity of the compounds towards the mentioned biomolecules [73,74,75]. The LGA method utilized for protein−ligand rigid−flexible docking included the following parameters: a maximum of 250,000 energy evaluations, 27,000 generations, and mutation and crossover rates of 0.02 and 0.8, respectively. This analysis included several steps, namely the ligand’s preparation, selection, protein preparation, and grid formation. The crystal structure of the HSA receptor complexed with warfarin (PDB:2BXD) was retrieved from RCSB Protein Data Bank in PDB with clearly defined active sites I (subdomain IIA). The PDB crystal structure of the HSA receptor with ibuprofen (2BXG) was used to determine active site II (subdomains IIIA) [76]. Active site II was defined when the 2BXD crystal structure was aligned to 2BXG by the USCF Chimera MatchMaker module. The crystal structure of BSA was obtained in the RCSB Protein Data Bank (PDB ID: 4F5S). The initial structure was changed in BIOVIA Discovery Studio 4.0 [77], and the chain B, residual atoms, heteroatoms, and water molecules were removed. The search space of BSA was restricted to a grid box size of 60 × 60 × 60 Å with a grid spacing of 0.375 A following the XYZ dimensions for site I (IIA): −4.80 × 30.50 ×101.01, and for site II (IIIA): 10.91 × 16.30 × 119.72. The initial structure of canonical B-DNA (PDB ID: 1BNA) and DNA with an intercalation gap (PDB ID: 1Z3F) were also obtained from the RCSB Protein Data Bank [78,79]. Grid box dimensions of 60 × 74 × 120 Å for the 1BNA structure were set at 15.81 × 21.31 × 9.88 Å with a grid spacing of 0.375 Å. The other parameters were the same as those used in previous research [80]. The AutoDock program calculates the free energy of binding values according to the following equation, Equation (3):

Δ***G***_bind_
*=* Δ***G***_vdw+hbond+desolv_ *+* Δ***G***_elec_ *+* Δ***G***_total_ *+* Δ***G***_tor_ − Δ***G***_unb_ (3)

where Δ***G***_bind_ is the estimated free energy of binding, and Δ***G***_vdw+hbond+desolv_ denotes the sum of the energies of dispersion and repulsion (Δ***G***_vdw_), hydrogen bond (Δ***G***_hbond_), and desolvation (Δ***G***_desolv_). Δ***G***_total_ represents the final total internal energy, Δ***G***_tor_ is torsional free energy, Δ***G***_unb_ is the unbound system’s energy, and Δ***G***_elec_ is electrostatic energy.

### 3.8. Cells

Human cell lines (A375 melanoma, MCF-7 breast cancer, HCT116 colorectal carcinoma, A2780 ovarian cancer, and MRC5 lung fibroblasts) were purchased from American Type Culture Collection (Rockville, MD, USA).

The A2780 cells were cultivated in DMEM, while all of the other cell lines were cultivated in the HEPES-buffered RPMI-1640 medium. The cell culture mediums were supplemented with penicillin (100 units/mL), streptomycin (100 μg/mL), and 10% inactivated FBS. All of the cells were cultivated at 37 °C in a humidified atmosphere with 5% CO_2_. 

### 3.9. Viability Assays (MTT and CV)

The cells were seeded overnight in 96-well plates: A2780 and MRC-5 8 × 10^3^ cells/well, HCT 116 6 × 10^3^ cells/well, A375 5 × 10^3^ cells/well, and MCF-7 at 10 × 10^3^ cells/well [58]. All of the cells were treated with the following concentrations of investigated compounds: 100, 50, 25, 12.5, 6.25, 3.125, and 1.56 μM, except for the A2780 cells, which were treated with the following concentrations for the Cu compound only: 25, 12.5, 6.25, 3.125, 1.56, 0.78, and 0.39 μM. After 72 h of treatment, viability assays were conducted.

## 4. Conclusions

A novel crystal structure of Ni-pyridoxal-thiosemicarbazone (Ni-PLTSC) was obtained and described. Hirshfeld analysis of the crystal structure of PLTSC and the corresponding Fe, Co, Ni, and Cu complexes proved the importance of geometry, other ligands, and solvent molecules for stabilizing the crystal structure. The percentage of specific contacts depended on the number of PLTSC units. The DFT analysis of the corresponding Fe-PLTSC, Co-PLTSC, and Cu-PLTSC showed that the theoretical structure represented the crystallographic one well, with the MAE value for the bond lengths being between 0.01 (Co-PLTSC) and 0.04 Å (Fe-PLTSC and Cu-PLTSC), while the MAE values for bond angles were between 1.2 (Co-PLTSC) and 2.9° (Cu-PLTSC). During the optimization process, the original octahedral structures were restored. The binding affinity towards Bovine Serum Albumin (BSA) and Humane Serum Albumin (HSA), investigated using spectrofluorometry, showed different behavior for the compounds, with the most stable structures being formed between Cu-PLTSC and transportation proteins (−42.5 (BSA) and −46.3 kJ mol^−1^ (HSA)). The molecular docking simulations towards these proteins outlined some limitations when the results were compared with the experimental ones. Overall, the same reactivity trend was obtained during the simulation and experiments. The DNA binding affinity was again the highest in the case of Cu-PLTSC. The cytotoxicity, investigated towards A375 melanoma, MCF-7 breast cancer, HCT116 colorectal carcinoma, and A2780 ovarian cancer, pointed out that Cu-PLTSC was the most active compound with micro-molar IC_50_ values determined by MTT and CV tests. This compound acted selectively towards A2780 when compared with the healthy lung fibroblasts, MRC5. The cytotoxic activity of Cu-PLTSC correlated well with the theoretically determined stability, reactivity, and possibility of interaction formation with the surrounding amino acids and nucleobases. Further biological studies are advised to determine the mechanism of cell death and further support these theoretical findings. 

## Figures and Tables

**Table 1:** Thermodynamic parameters of binding of investigated complexes to BSA at different temperatures.

Compound	T [K]	K_b_ [M^−1^]	n	R^2^	ΔH_b_ [kJ mol^−1^]	ΔS_b_ [J mol^−1^ K^−1^]	ΔG_b_ [kJ mol^−1^]
**Fe-PLTSC**	300	1.32 × 10^5^	1.1	0.997	15.5	149	−29.4
	305	1.48 × 10^5^	1.1	0.997			−30.2
	310	1.61 × 10^5^	1.1	0.993			−30.9
**Co-PLTSC**	300	2.14 × 10^5^	1.1	0.986	70.3	33.7	−30.7
	305	4.07 × 10^5^	1.1	0.999			−32.4
	310	5.29 × 10^5^	1.1	0.997			−34.1
**Ni-PLTSC**	300	1.71 × 10^7^	1.2	0.962	−79.4	−126	−41.7
	305	1.11 × 10^7^	1.2	0.996			−41.0
	310	6.13 × 10^6^	1.2	0.991			−40.4
**Cu-PLTSC**	300	6.24 × 10^6^	1.3	0.996	67.4	355	−38.9
	305	9.19 × 10^6^	1.2	0.997			−40.7
	310	1.49 × 10^7^	1.2	0.995			−42.5

**Table 2:** Thermodynamic parameters of binding of investigated complexes to HSA at different temperatures.

Compound	T [K]	K_b_ [M^−1^]	n	R^2^	ΔH_b_ [kJ mol^−1^]	ΔS_b_ [J mol^−1^ K^−1^]	ΔG_b_ [kJ mol^−1^]
**Fe-PLTSC**	300	7.55 × 10^5^	1.2	0.999	58.3	307	−33.8
	305	1.26 × 10^6^	1.2	0.999			−35.4
	310	1.65 × 10^6^	1.1	0.998			−36.9
**Co-PLTSC**	300	8.07 × 10^4^	1.0	0.999	12.7	136	−28.2
	305	8.79 × 10^4^	1.0	0.996			−28.9
	310	9.50 × 10^4^	1.0	0.999			−29.5
**Ni-PLTSC**	300	2.67 × 10^6^	1.2	0.991	−31.4	15.5	−37.1
	305	2.57 × 10^6^	1.2	0.993			−37.2
	310	1.75 × 10^6^	1.2	0.994			−37.2
**Cu-PLTSC**	300	3.04 × 10^7^	1.2	0.991	69.1	372	−42.6
	305	3.40 × 10^7^	1.2	0.993			−44.5
	310	7.46 × 10^7^	1.2	0.994			−46.3

**Table 3:** Important thermodynamic parameters (ΔG_bind_ free binding energy, K_i_ constant of inhibition, ΔG_total_ final total internal energy, ΔG_tor_ torsional free energy, ΔG_unb_ unbound system’s energy, ΔG_elec_ electrostatic energy and $\Delta G_{\mathrm{vdw}+\mathrm{hbond}+\mathrm{desolv}}$ is the sum of dispersion and repulsion (ΔG_vdw_), hydrogen bond (ΔG_hbond_), and desolvation (ΔG_desolv_) energy predicted for most stable docking conformations.

Conformation	ΔG*_bind_*	K_i_ (µM)	ΔG*_inter_*	ΔG*_vdw+hbond+desolv_*	ΔG*_elec_*	ΔG*_total_*	ΔG*_tor_*	ΔG*_unb_*
**BSA-Cu-PLTSC-IIA**	−22.8	99.2	−27.4	−27.0	−0.5	−2.2	4.6	−2.2
**BSA-Cu-PLTSC-IIIA**	−24.6	48.8	−29.2	−29.0	−0.2	−2.1	4.6	−2.1
**BSA-Fe-PLTSC-IIA**	−24.4	53.1	−29.0	−28.6	−0.4	−1.5	4.6	−1.5
**BSA-Fe-PLTSC-IIIA**	−20.3	0.3	−24.9	−23.7	−1.2	−1.4	4.6	−1.4
**BSA-Co-PLTSC-IIA**	−19.9	319.2	−26.8	−25.8	−1.0	1.0	6.9	1.0
**BSA-Co-PLTSC-IIIA**	−17.0	0.1	−24.5	−21.5	−3.0	1.0	7.5	1.0
**BSA-Ni-PLTSC-IIA**	−20.5	0.25	−25.1	−23.5	−1.6	0.1	4.6	0.1
**BSA-Ni-PLTSC-IIIA**	−18.0	699.9	−22.6	−21.7	−0.9	0.2	4.6	0.2

**Table 4:** IC_50_ values (in μM) for the cytotoxicity studies on the investigated complexes.

Cell Line		Assay	Fe-PLTSC	Co-PLTSC	Cu-PLTSC	Ni-PLTSC
HCT116	Colorectal carcinoma	MTT	>100	>100	17.5 ± 1.8	>100
		CV	>100	>100	18.9 ± 0.6	>100
A375	melanoma	MTT	>100	>100	74.6 ± 2.3	>100
		CV	>100	>100	98.7 ± 1.9	>100
MCF-7	Breast cancer	MTT	>100	>100	25.4 ± 0.6	>100
		CV	>100	>100	41.3 ± 3.3	>100
A2780	Ovarian cancer	MTT	86.3 ± 13.1	>100	1.8 ± 0.1	>100
		CV	93.5 ± 9.2	>100	1.7 ± 0.1	>100
MRC5	Lung fibroblasts	MTT	>100	>100	4.9 ± 0.1	>100
		CV	>100	>100	5.2 ± 0.4	>100

**Table 5:** Crystallographic data of the newly obtained structure of Ni-PLTSC.

Empirical Formula	C_18_H_26_N_10_NiO_11_S_2_
Molecular mass	681.32
Crystal system	triclinic
Space group	P-1
a (A)	8.0150 (2)
b (A)	10.9287 (3)
c (A)	16.5250 (4)
α (°)	102.013 (2)
β (°)	98.858 (2)
Γ (°)	105.418 (2)
V (A^3^)	1330.78 (6)
Z	2
F(000)	704.0
Dx, g cm^−3^	1.700
Theta (max)	79.852
hkl Ranges	10, 13, 21
T (K)	173

## Data Availability

Not applicable.

## Supplementary Materials

The following supporting information can be downloaded at: https://www.mdpi.com/article/10.3390/ijms241511910/s1.

## References

[1] Oun R., Moussa Y.E., Wheate N.J. (2018). The side effects of platinum-based chemotherapy drugs: A review for chemists. Dalt. Trans..

[2] Yu C., Wang Z., Sun Z., Zhang L., Zhang W., Xu Y., Zhang J.-J. (2020). Platinum-Based Combination Therapy: Molecular Rationale, Current Clinical Uses, and Future Perspectives. J. Med. Chem..

[3] Jamieson E.R., Lippard S.J. (1999). Structure, Recognition, and Processing of Cisplatin−DNA Adducts. Chem. Rev..

[4] Todd R.C., Lippard S.J. (2009). Inhibition of transcription by platinum antitumor compounds. Metallomics.

[5] Wang D., Lippard S.J. (2005). Cellular processing of platinum anticancer drugs. Nat. Rev. Drug Discov..

[6] Eichhorn T., Kolbe F., Mišić S., Dimić D., Morgan I., Saoud M., Milenković D., Marković Z., Rüffer T., Dimitrić Marković J. (2023). Synthesis, Crystallographic Structure, Theoretical Analysis, Molecular Docking Studies, and Biological Activity Evaluation of Binuclear Ru(II)-1-Naphthylhydrazine Complex. Int. J. Mol. Sci..

[7] Lobana T.S., Sharma R., Bawa G., Khanna S. (2009). Bonding and structure trends of thiosemicarbazone derivatives of metals—An overview. Coord. Chem. Rev..

[8] Ozturk I.I. (2023). Synthesis, Characterization and Hirshfeld Surface Analysis of Some Thiosemicarbazones Containing a Five-Membered Ring. J. Struct. Chem..

[9] Prajapati N.P., Patel H.D. (2019). Novel thiosemicarbazone derivatives and their metal complexes: Recent developments. Synth. Commun..

[10] Venkatraman R., Ameera H., Sitole L., Ellis E., Fronczek F.R., Valente E.J. (2009). Structures of Eight Thio(semi)carbazones Derived from 2-Acetylpyrazine, 2-Acetythiazole and Acetophenone. J. Chem. Crystallogr..

[11] Casas J.S., García-Tasende M.S., Sordo J. (2000). Main group metal complexes of semicarbazones and thiosemicarbazones. A structural review. Coord. Chem. Rev..

[12] Rani M., Devi J., Kumar B. (2023). Thiosemicarbazones—Based Co (II), Ni (II), Cu (II) and Zn (II) complexes: Synthesis, structural elucidation, biological activities and molecular docking. Chem. Pap..

[13] Tido E.W.Y., Faulmann C., Roswanda R., Meetsma A., van Koningsbruggen P.J. (2010). Tuning of the charge in octahedral ferric complexes based on pyridoxal-N-substituted thiosemicarbazone ligands. Dalt. Trans..

[14] Lobana T.S., Butcher R.J. (2004). Metal—Thiosemicarbazone interactions. Synthesis of an iodo-bridged dinuclear [diiodobis (pyrrole-2-carbaldehydethiosemicarbazone) dicopper (I)] complex. Transit. Met. Chem..

[15] Hidalgo T., Fabra D., Allende R., Matesanz A.I., Horcajada P., Biver T., Quiroga A.G. (2023). Two novel Pd thiosemicarbazone complexes as efficient and selective antitumoral drugs. Inorg. Chem. Front..

[16] Minh Quang N., Tran Thai H., Le Thi H., Duc Cuong N., Hien N.Q., Hoang D., Ngoc V.T.B., Ky Minh V., Van Tat P. (2023). Novel Thiosemicarbazone Quantum Dots in the Treatment of Alzheimer’s Disease Combining In Silico Models Using Fingerprints and Physicochemical Descriptors. ACS Omega.

[17] Khan M., Gohar H., Alam A., Wadood A., Shareef A., Ali M., Khalid A., Abdalla A.N., Ullah F. (2023). Para-Substituted Thiosemicarbazones as Cholinesterase Inhibitors: Synthesis, In Vitro Biological Evaluation, and In Silico Study. ACS Omega.

[18] Belicchi-Ferrari M., Bisceglie F., Casoli C., Durot S., Morgenstern-Badarau I., Pelosi G., Pilotti E., Pinelli S., Tarasconi P. (2005). Copper(II) and Cobalt(III) Pyridoxal Thiosemicarbazone Complexes with Nitroprusside as Counterion: Syntheses, Electronic Properties, and Antileukemic Activity. J. Med. Chem..

[19] Leovac V.M., Jovanović L.S., Jevtović V.S., Pelosi G., Bisceglie F. (2007). Transition metal complexes with thiosemicarbazide-based ligand—Part LV: Synthesis and X-ray structural study of novel Ni(II) complexes with pyridoxal semicarbazone and pyridoxal thiosemicarbazone. Polyhedron.

[20] Leovac V.M., Jevtović V.S., Jovanović L.S., Bogdanović G.A. (2005). Metal complexes with schiff-base ligands—Pyridoxal and semicarbazide-based derivatives. J. Serbian Chem. Soc..

[21] Aly A.A., Abdallah E.M., Ahmed S.A., Rabee M.M., Bräse S. (2023). Transition Metal Complexes of Thiosemicarbazides, Thiocarbohydrazides, and Their Corresponding Carbazones with Cu(I), Cu(II), Co(II), Ni(II), Pd(II), and Ag(I)—A Review. Molecules.

[22] Lobana T.S., Bawa G., Butcher R.J., Liaw B.J., Liu C.W. (2006). Thiosemicarbazonates of ruthenium(II): Crystal structures of [bis(diphenylphosphino)butane][bis(pyridine-2-carbaldehydethiosemicarbazonato)] ruthenium(II) and [bis(triphenylphosphine)][bis(benzaldehydethiosemicarbazonato)] ruthenium(II). Polyhedron.

[23] Fuior A., Cebotari D., Garbuz O., Calancea S., Gulea A., Floquet S. (2023). Biological properties of a new class of [Mo_2_O_2_S_2_]-based thiosemicarbazone coordination complexes. Inorganica Chim. Acta.

[24] Ali M.S., El-Saied F.A., Shakdofa M.M., Karnik S., Jaragh-Alhadad L.A. (2023). Synthesis and characterization of thiosemicarbazone metal complexes: Crystal structure, and antiproliferation activity against breast (MCF7) and lung (A549) cancers. J. Mol. Struct..

[25] Savir S., Wei Z.J., Liew J.W.K., Vythilingam I., Lim Y.A.L., Saad H.M., Sim K.S., Tan K.W. (2020). Synthesis, cytotoxicity and antimalarial activities of thiosemicarbazones and their nickel (II) complexes. J. Mol. Struct..

[26] Leovac V.M., Jevtović V.S., Bogdanovic G.A. (2002). Transition metal complexes with thio-semicarbazide-based ligands. XLIV1. Aqua(3-hydroxy-5-hydroxymethyl-2-methylpyridine-4- carboxaldehyde 3-methylisothiosemicarbazone-κ3O, N1, N4)nitratocopper(II) nitrate. Acta Crystallogr. Sect. C Cryst. Struct. Commun..

[27] Rodić M.V., Radanović M.M., Vojinović-Ješić L.S., Belošević S.K., Jaćimović Ž.K., Leovac V.M. (2019). Synthesis and crystal structure of copper(II) complexes with pyridoxal S-methylisothiosemicarbazone bearing a new coordination mode. J. Serbian Chem. Soc..

[28] Ivković S.A., Vojinović-Ješić L.S., Leovac V.M., Rodić M.V., Novaković S.B., Bogdanović G.A. (2015). Transition metal complexes with thiosemicarbazide-based ligands. Part 61. Comparative analysis of structural properties of the pyridoxal thiosemicarbazone ligands. Crystal structure of PLTSC·HCl·2H_2_O and its complex [Fe(PLTSC)Cl_2_(H_2_O)]Cl. Struct. Chem..

[29] Altamimi A.S., Al-zahrani S.A., Jevtovic V. (2021). Synthesis, Structure Analysis and Antibacterial Activity of Zn (II) and Co (III) Complexes. Am. J. Chem..

[30] Avdović E.H., Milanović Ž.B., Molčanov K., Roca S., Vikić-Topić D., Mrkalić E.M., Jelić R.M., Marković Z.S. (2022). Synthesis, characterization and investigating the binding mechanism of novel coumarin derivatives with human serum albumin: Spectroscopic and computational approach. J. Mol. Struct..

[31] Fasano M., Curry S., Terreno E., Galliano M., Fanali G., Narciso P., Notari S., Ascenzi P. (2005). The extraordinary ligand binding properties of human serum albumin. IUBMB Life (Int. Union Biochem. Mol. Biol. Life).

[32] Fanali G., di Masi A., Trezza V., Marino M., Fasano M., Ascenzi P. (2012). Human serum albumin: From bench to bedside. Mol. Aspects Med..

[33] Liu T., Liu M., Guo Q., Liu Y., Zhao Y., Wu Y., Sun B., Wang Q., Liu J., Han J. (2020). Investigation of binary and ternary systems of human serum albumin with oxyresveratrol/piceatannol and/or mitoxantrone by multipectroscopy, molecular docking and cytotoxicity evaluation. J. Mol. Liq..

[34] Chugh H., Kumar P., Tomar V., Kaur N., Sood D., Chandra R. (2019). Interaction of noscapine with human serum albumin (HSA): A spectroscopic and molecular modelling approach. J. Photochem. Photobiol. A Chem..

[35] Bagheri M., Fatemi M.H. (2018). Fluorescence spectroscopy, molecular docking and molecular dynamic simulation studies of HSA-Aflatoxin B1 and G1 interactions. J. Lumin..

[36] Palchaudhuri R., Hergenrother P.J. (2007). DNA as a target for anticancer compounds: Methods to determine the mode of binding and the mechanism of action. Curr. Opin. Biotechnol..

[37] Vojinović-Ješić L.S., Rodić M.V., Barta Holló B., Ivković S.A., Leovac V.M., Mészáros Szécsényi K. (2016). Synthesis, characterization and thermal behavior of copper(II) complexes with pyridoxal thiosemi (PLTSC)- and S-methylisothiosemicarbazone (PLITSC). J. Therm. Anal. Calorim..

[38] Danac R., Pui A., Corja I., Amarandi R.M., Ciobanu C.I., Apostu M.O., Palamarciuc O. (2020). New M(II) (M=Mn, Co, Ni, Cu, Zn, Pd) coordinative compounds with 2-formylpyridine S-methyl-isothiosemicarbazide. J. Mol. Struct..

[39] Graur V., Chumakov Y., Garbuz O., Hureau C., Tsapkov V., Gulea A. (2022). Synthesis, Structure, and Biologic Activity of Some Copper, Nickel, Cobalt, and Zinc Complexes with 2-Formylpyridine N4-Allylthiosemicarbazone. Bioinorg. Chem. Appl..

[40] Gak Simić K., Đorđević I., Lazić A., Radovanović L., Petković-Benazzouz M., Rogan J., Trišović N., Janjić G. (2021). On the supramolecular outcomes of fluorination of cyclohexane-5-spirohydantoin derivatives. Cryst. Eng. Comm..

[41] Janjić G.V., Jelić S.T., Trišović N.P., Popović D.M., Dordević I.S., Milčić M.K. (2020). New Theoretical Insight into Fluorination and Fluorine-Fluorine Interactions as a Driving Force in Crystal Structures. Cryst. Growth Des..

[42] Jevtović V., Hamoud H., Al-zahrani S., Alenezi K., Latif S., Alanazi T., Abdulaziz F., Dimić D. (2022). Synthesis, Crystal Structure, Quantum Chemical Analysis, Electrochemical Behavior, and Antibacterial and Photocatalytic Activity of Co Complex with. Molecules.

[43] Jevtovic V., Alshammari N., Latif S., Alsukaibi A.K.D., Humaidi J., Alanazi T.Y.A., Abdulaziz F., Matalka S.I., Pantelić N.Đ., Marković M. (2022). Synthesis, Crystal Structure, Theoretical Calculations, Antibacterial Activity, Electrochemical Behavior, and Molecular Docking of Ni(II) and Cu(II) Complexes with Pyridoxal-Semicarbazone. Molecules.

[44] Aly S.A., Eldourghamy A., El-Fiky B.A., Megahed A.A., El-Sayed W.A., Abdalla E.M., Elganzory H.H. (2022). Synthesis, spectroscopic characterization, thermal studies, and molecular docking of novel Cr(III), Fe(III), and Co(II) complexes based on Schiff base: In vitro antibacterial and antitumor activities. J. Appl. Pharm. Sci..

[45] Ibrahim M.M., El-Kemary M.A., Al-Harbi S.A., Al-Saidi H.M., Sallam S.A., Ramadan A.E.-M.M. (2021). Synthesis and structural characterization of Pyridine-based Mn(II), Fe(III), and Co(III) complexes as SOD mimics and BSA binding studies. J. Mol. Struct..

[46] Rudra S., Dasmandal S., Patra C., Kundu A., Mahapatra A. (2016). Binding affinities of Schiff base Fe(II) complex with BSA and calf-thymus DNA: Spectroscopic investigations and molecular docking analysis. Spectrochim. Acta-Part A Mol. Biomol. Spectrosc..

[47] Guo J.-L., Liu G.-Y., Wang R.-Y., Sun S.-X. (2022). Synthesis and structure elucidation of two essential metal complexes: In-vitro studies of their BSA/HSA-binding properties, docking simulations, and anticancer activities. Molecules.

[48] Zhang Y.-Z., Li H.-R., Dai J., Chen W.-J., Zhang J., Liu Y. (2010). Spectroscopic studies on the binding of cobalt(II) 1,10-phenanthroline complex to Bovine Serum Albumin. Biol. Trace Elem Res..

[49] Ray A., Seth B.K., Pal U., Basu S. (2012). Nickel(II)-Schiff base complex recognizing domain II of bovine and humane serum albumin: Spectroscopic and docking studies. Spectrochim. Acta-Part A Mol. Biomol. Spectrosc..

[50] Duru Kamaci U., Kamaci M., Peksel A. (2017). Thermally Stable Schiff Base and its Metal Complexes: Molecular Docking and Protein Binding Studies. J. Fluoresc..

[51] Bera M., Das M., Dolai M., Laha S., Islam M.M., Samanta B.C., Das A., Choudhuri I., Bhattacharyya N., Maity T. (2023). DNA/Protein Binding and Apoptotic-Induced Anticancer Property of a First Time Reported Quercetin–Iron(III) Complex Having a Secondary Anionic Residue: A Combined Experimental and Theoretical Approach. ACS Omega.

[52] Bai H., Shi J., Guo Q., Wang W., Zhang Z., Li Y., Vennampalli M., Zhao X., Wang H. (2022). Spectroscopy, Structure, Biomacromolecular Interactions, and Antiproliferation Activity of a Fe(II) Complex With DPA-Bpy as Pentadentate Ligand. Front. Chem..

[53] De Simone G., di Masi A., Ascenzi P. (2021). Serum Albumin: A Multifaced Enzyme. Int. J. Mol. Sci..

[54] Behnamfar M.T., Hadadzadeh H., Simpson J., Darabi F., Shahpiri A., Khayamian T., Ebrahimi M., Amiri Rudbari H., Salimi M. (2015). Experimental and molecular modeling studies of the interaction of the polypyridyl Fe(II) and Fe(III) complexes with DNA and BSA. Spectrochim. Acta-Part A Mol. Biomol. Spectrosc..

[55] Li Y., Qian C., Li Y., Yang Y., Lin D., Liu X., Chen C. (2021). Syntheses, crystal structures of two Fe(III) Schiff base complexes with chelating o-vanillin aroylhydrazone and exploration of their bio-relevant activities. J. Inorg. Biochem..

[56] Hassani Moghadam F., Taher M.A., Karimi-Maleh H. (2021). Doxorubicin Anticancer Drug Monitoring by ds-DNA-Based Electrochemical Biosensor in Clinical Samples. Micromachines.

[57] Shamsi F., Aneja B., Hasan P., Zeya B., Zafaryab M., Mehdi S.H., Rizvi M.M.A., Patel R., Rana S., Abid M. (2019). Synthesis, Anticancer Evaluation and DNA-Binding Spectroscopic Insights of Quinoline-Based 1,3,4-Oxadiazole-1,2,3-triazole Conjugates. Chem. Sel..

[58] Predarska I., Saoud M., Drača D., Morgan I., Komazec T., Eichhorn T., Mihajlović E., Dunđerović D., Mijatović S., Maksimović-Ivanić D. (2022). Mesoporous Silica Nanoparticles Enhance the Anticancer Efficacy of Platinum(IV)-Phenolate Conjugates in Breast Cancer Cell Lines. Nanomaterials.

[59] (2017). CrysAlisPRO.

[60] Turner M.J., McKinnon J.J., Wolff S.K., Grimwood D.J., Spackman P.R., Jayatilaka D., Spackman M.A. (2017). CrystalExplorer17.

[61] Karrouchi K., Brandán S.A., Sert Y., El Karbane M., Radi S., Ferbinteanu M., Garcia Y., Ansar M. (2021). Synthesis, structural, molecular docking and spectroscopic studies of (E)-N′-(4-methoxybenzylidene)-5-methyl-1H-pyrazole-3-carbohydrazide. J. Mol. Struct..

[62] Spackman M.A., Jayatilaka D. (2009). Hirshfeld surface analysis. CrystEngComm.

[63] Spackman M.A., Byrom P.G. (1997). A novel definition of a molecule in a crystal. Chem. Phys. Lett..

[64] Frisch M.J., Trucks G.W., Schlegel J., Scuseria G.E., Robb M.A., Cheeseman J.R., Schlegel H.B., Scalmani G., Barone V., Mennucci B. (2009). Gaussian 09, Revision C.01.

[65] Becke A.D. (1993). Density-functional thermochemistry. III. The role of exact exchange. J. Chem. Phys..

[66] Dunning T.H. (1989). Gaussian basis sets for use in correlated molecular calculations. I. The atoms boron through neon and hydrogen. J. Chem. Phys..

[67] Hay P.J., Wadt W.R. (1985). Ab initio effective core potentials for molecular calculations. Potentials for the transition metal atoms Sc to Hg. J. Chem. Phys..

[68] Hay P.J., Wadt W.R. (1985). Ab initio effective core potentials for molecular calculations. Potentials for K to Au including the outermost core orbitale. J. Chem. Phys..

[69] Dennington R., Todd K., Millam J. (2009). GausView 2009.

[70] Reed A.E., Weinstock R.B., Weinhold F. (1985). Natural population analysis. J. Chem. Phys..

[71] Behjatmanesh-Ardakani R. (2015). NBO–NEDA, NPA, and QTAIM studies on the interactions between aza-, diaza-, and triaza-12-crown-4 (An-12-crown-4, n=1, 2, 3) and Li+, Na+, and K+ ions. Comput. Theor. Chem..

[72] Valdés-Tresanco M.S., Valdés-Tresanco M.E., Valiente P.A., Moreno E. (2020). AMDock: A versatile graphical tool for assisting molecular docking with Autodock Vina and Autodock4. Biol. Direct.

[73] Wang J., Wolf R.M., Caldwell J.W., Kollman P.A., Case D.A. (2004). Development and testing of a general amber force field. J. Comput. Chem..

[74] Milanović Ž.B., Antonijević M.R., Amić A.D., Avdović E.H., Dimić D.S., Milenković D.A., Marković Z.S. (2021). Inhibitory activity of quercetin, its metabolite, and standard antiviral drugs towards enzymes essential for SARS-CoV-2: The role of acid–base equilibria. RSC Adv..

[75] Amić A., Dimitrić Marković J.M., Marković Z., Milenković D., Milanović Ž., Antonijević M., Mastiľák Cagardová D., Rodríguez-Guerra Pedregal J. (2021). Theoretical Study of Radical Inactivation, LOX Inhibition, and Iron Chelation: The Role of Ferulic Acid in Skin Protection against UVA Induced Oxidative Stress. Antioxidants.

[76] Ghuman J., Zunszain P.A., Petitpas I., Bhattacharya A.A., Otagiri M., Curry S. (2005). Structural Basis of the Drug-binding Specificity of Human Serum Albumin. J. Mol. Biol..

[77] Dassault Systèmes BIOVIA (2016). Discovery Studio Modeling Environment (Release 2017).

[78] Canals A., Purciolas M., Aymamí J., Coll M. (2005). The anticancer agent ellipticine unwinds DNA by intercalative binding in an orientation parallel to base pairs. Acta Crystallogr. Sect. D Biol. Crystallogr..

[79] Drewt H.R., Wingtt R.M., Takanot T., Brokat C., Tanakat S., Itakuraii K., Dickersont R.E. (1981). Structure of a B-DNA dodecamer: Conformation and dynamics. Proc. Natl. Acad. Sci. USA.

[80] Dimić D.S., Kaluđerović G.N., Avdović E.H., Milenković D.A., Živanović M.N., Potočňák I., Samoľová E., Dimitrijević M.S., Saso L., Marković Z.S. (2022). Synthesis, Crystallographic, Quantum Chemical, Antitumor, and Molecular Docking/Dynamic Studies of 4-Hydroxycoumarin-Neurotransmitter Derivatives. Int. J. Mol. Sci..

