# Supplementary material for: The Effect of Metal Ions (Fe, Co, Ni, and Cu) on the Molecular-Structural, Protein Binding, and Cytotoxic Properties of Metal Pyridoxal-Thiosemicarbazone Complexes
Source: Int J Mol Sci. 2023 Jul 25;24(15):11910. doi: 10.3390/ijms241511910 (PMC10419307; doi:10.3390/ijms241511910)
Supplement: Supplementary file 1 [file ijms-24-11910-s001.zip › ijms-2508226-supplementary.pdf]

International Journal of *Molecular Sciences*

Supplementary Information

The Effect of Metal Ions (Fe, Co, Ni, and Cu) on the Molecular-Structural, Protein Binding, and Cytotoxic Properties of Metal Pyridoxal-Thiosemicarbazone Complexes

Violeta Jevtovic [1,*], Asma K. Alshamari [1], Dejan Milenković [2], Jasmina Dimitrić Marković [3], Zoran Marković [2] and Dušan Dimić [3,*]

[1] Department of Chemistry, College of Science, University Ha’il, Ha’il 81451, Saudi Arabia; ak.alshamari@uoh.edu.sa
[2] Department of Science, Institute for Information Technologies, University of Kragujevac, Jovana Cvijića bb, 34000 Kragujevac, Serbia; dejanm@uni.kg.ac.rs (D.M.); zmarkovic@uni.kg.ac.rs (Z.M.)
[3] Faculty of Physical Chemistry, University of Belgrade, Studentski trg 12-16, 11000 Belgrade, Serbia; markovich@ffh.bg.ac.rs
* Correspondence: v.jevtovic@uoh.edu.sa (V.J.) and ddimic@ffh.bg.ac.rs (D.D.)

Citation: Jevtovic, V.; Alshamari, A.K.; Milenković, D.; Dimitrić Marković, J.; Marković, Z.; Dimić, D. The Effect of Metal Ions (Fe, Co, Ni, and Cu) on the Molecular-Structural, Protein Binding, and Cytotoxic Properties of Metal Pyridoxal-Thiosemicarbazone Complexes. *Int. J. Mol. Sci.* **2023**, *24*, 1910. https://doi.org/10.3390/ijms241511910

Academic Editors: Claudia Riccardi, Marialuisa Piccolo

Received: 30 June 2023
Revised: 20 July 2023
Accepted: 21 July 2023
Published: 25 July 2023

Copyright: © 2023 by the authors. Licensee MDPI, Basel, Switzerland. This article is an open access article distributed under the terms and conditions of the Creative Commons Attribution (CC BY) license (https://creativecommons.org/licenses/by/4.0/).

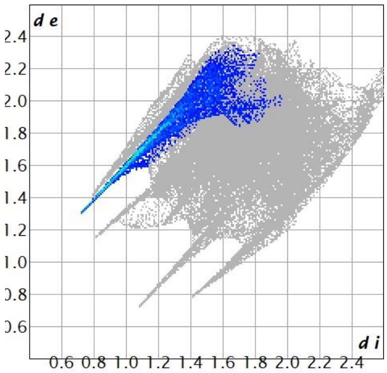

Cl···H (9.9%)

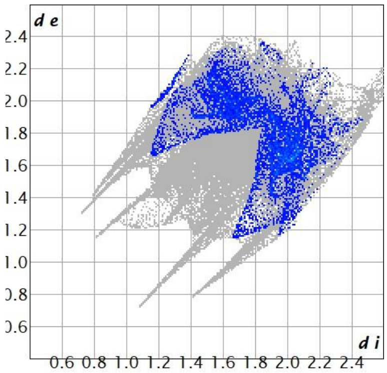

H···C (10.7%)

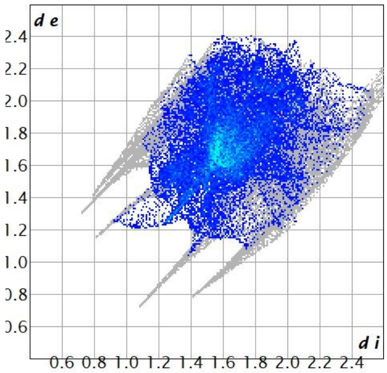

H···H (34.1%)

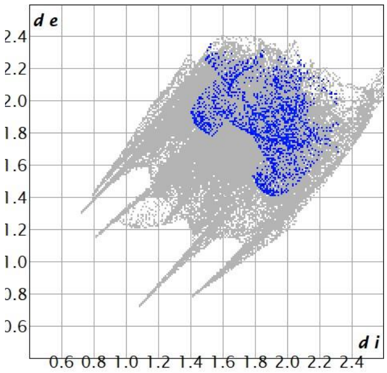

N···H (3.0%)

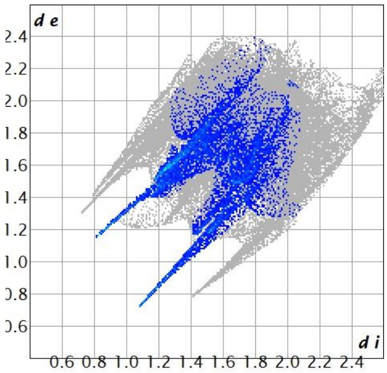

O···H (13.7%)

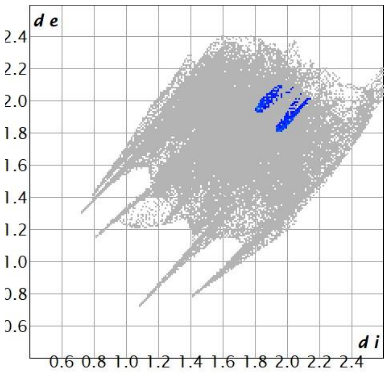

S···C (0.8%)

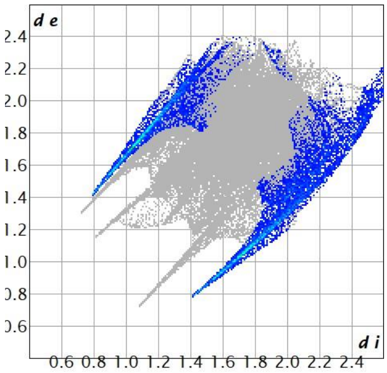

S···H (15.5%)

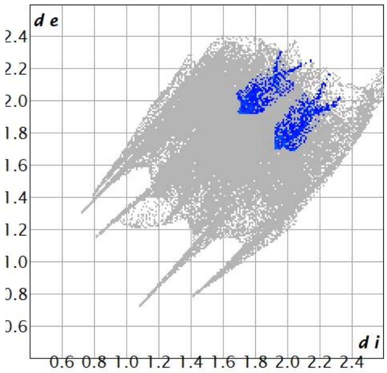

S···N (2.5%)

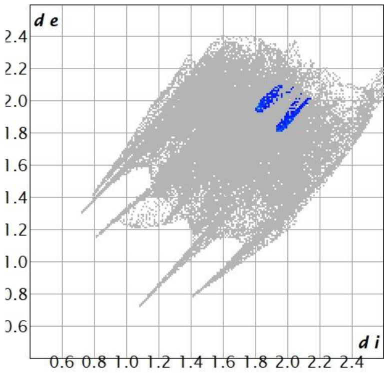

S···C (0.8%)

Figure S1. The fingerprint plots of the selected contacts within PLTSC structure

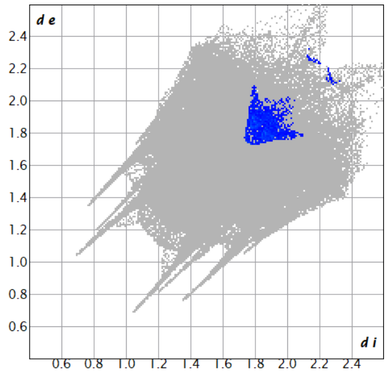

C···C (1.5%)

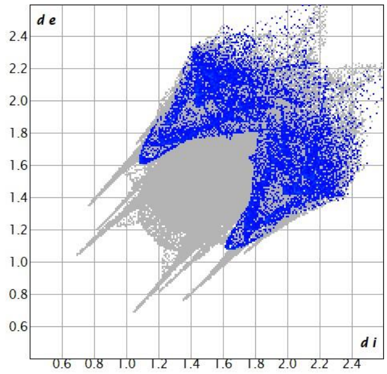

C···H (8.7%)

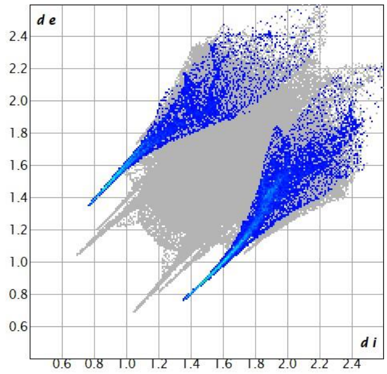

Cl···H (11.4%)

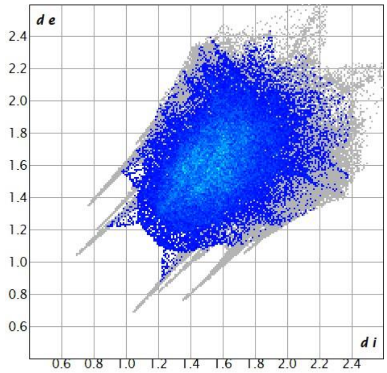

H···H (40.4%)

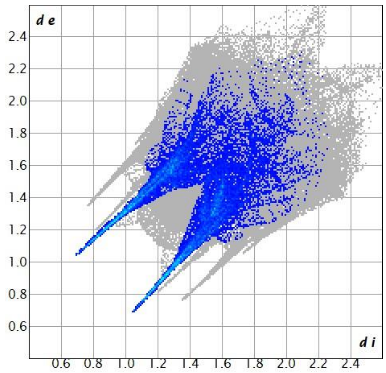

H···O (15.5%)

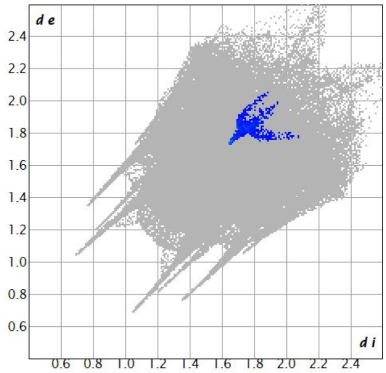

N···C (1.5%)

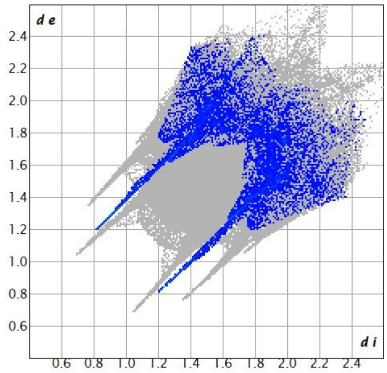

N···H (8.6%)

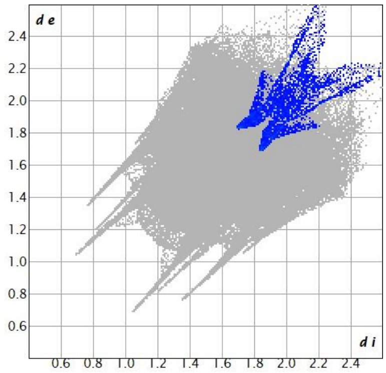

S···C (3.0%)

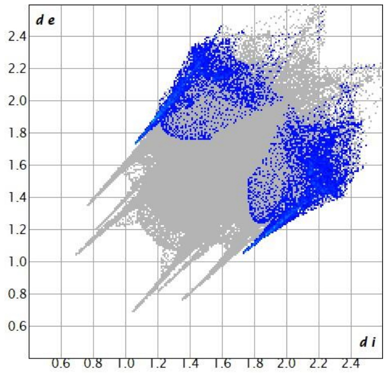

S···H (7.6%)

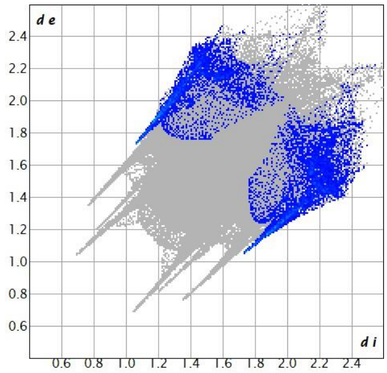

S···N (0.9%)

Figure S2. The fingerprint plots of the selected contacts within Co-PLTSC structure

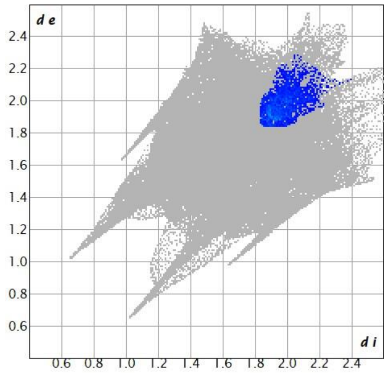

C···C (3.0%)

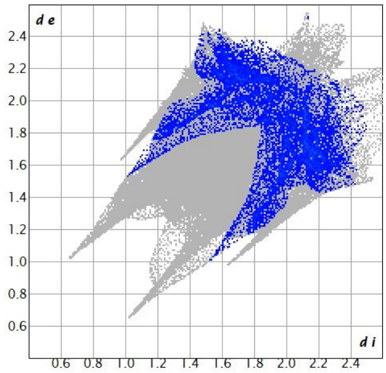

H···C (9.8%)

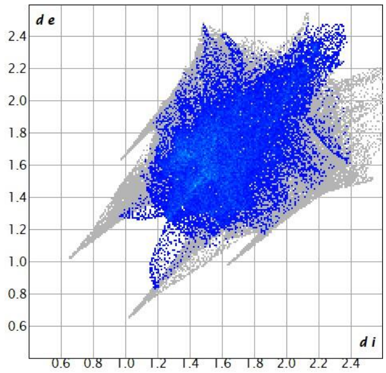

H···H (26.2%)

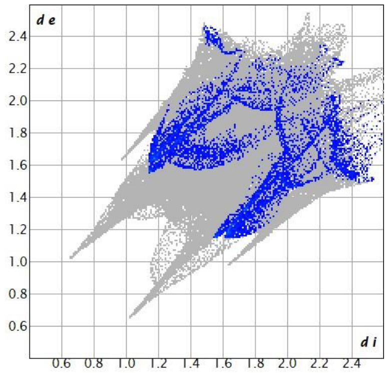

N···H (5.3%)

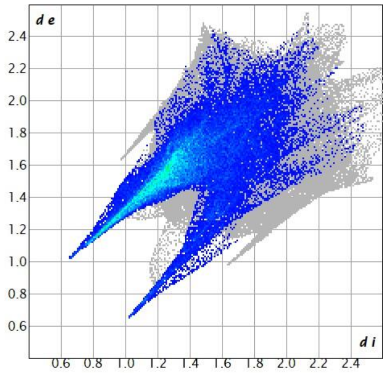

O···H (37.6%)

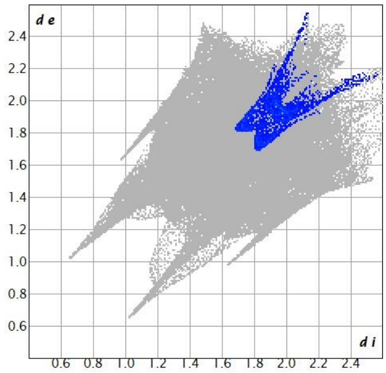

S···C (2.9%)

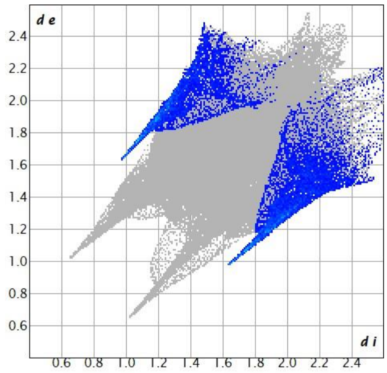

S···H (10.4%)

Figure S3. The fingerprint plots of the selected contacts within Co-PLTSC structure

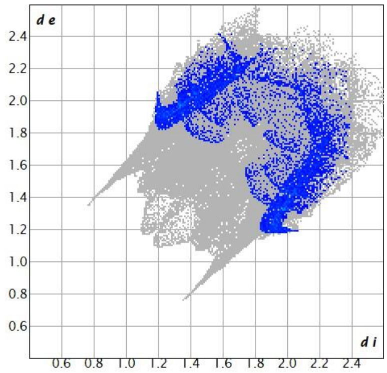

C···H (8.9%)

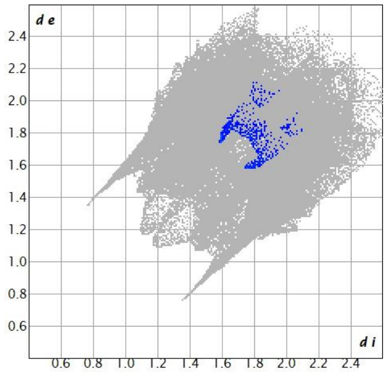

N···C (0.8%)

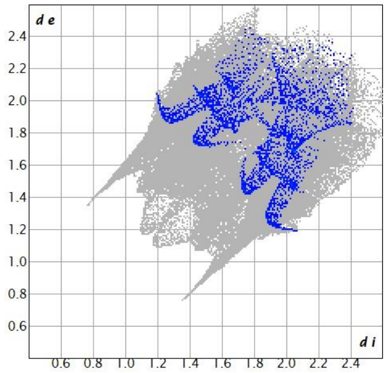

N···H (3.6%)

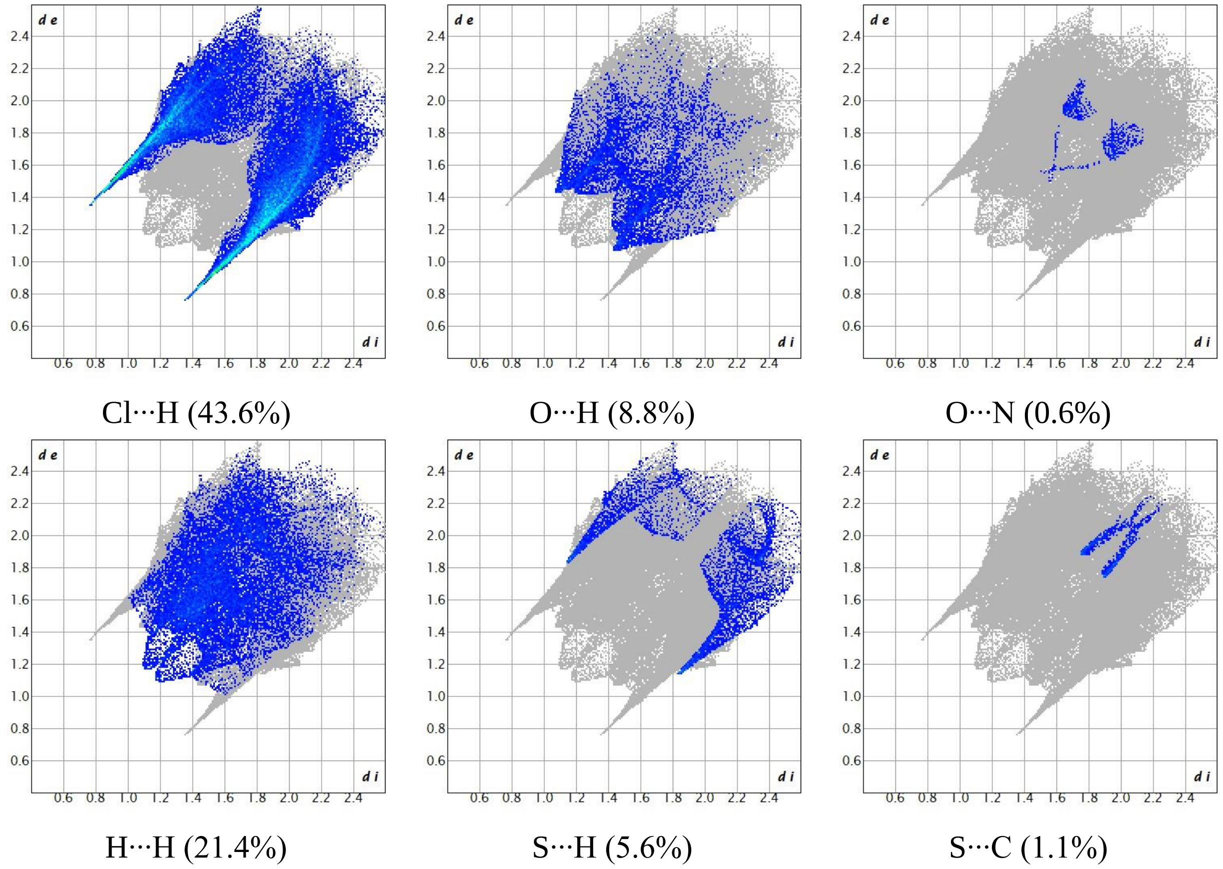

Figure S4. The fingerprint plots of the selected contacts within Co-PLTSC structure

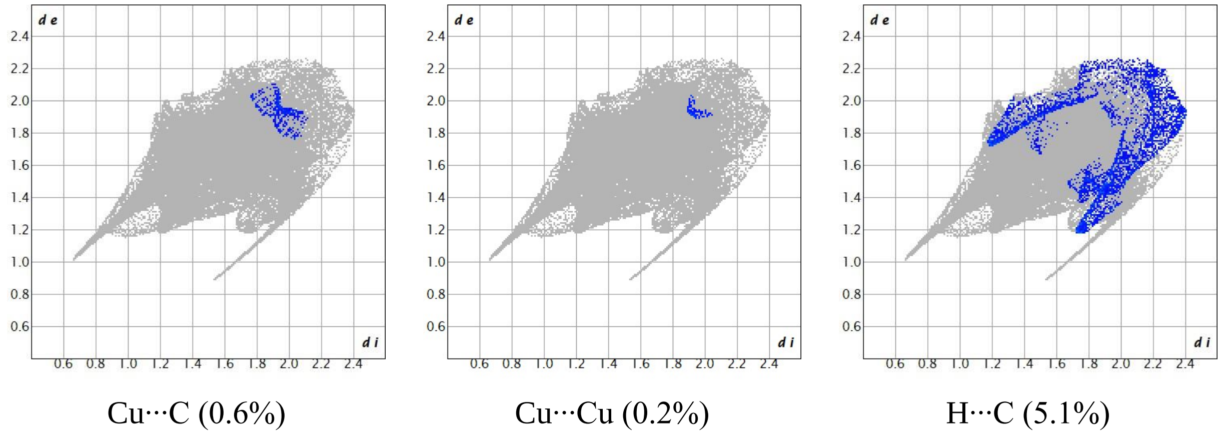

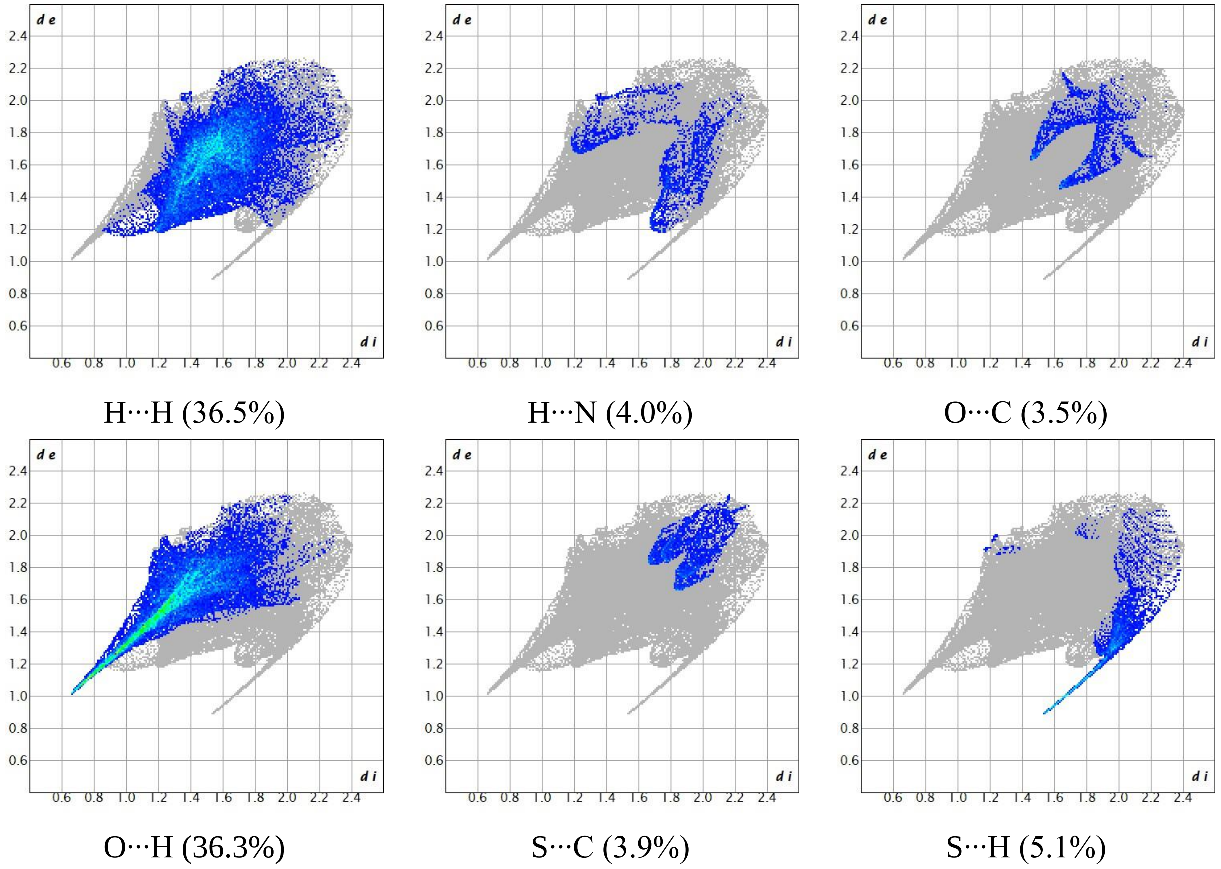

Figure S5. The fingerprint plots of the selected contacts within Co-PLTSC structure

Table S1. Crystallographic and optimized (at B3LYP/6-31+G(d,p) level of theory bond lengths (in Å) of PLTSC.

Bond	Optimized	Experimental
O22-C6	1.34	1.34
C6-C11	1.40	1.40
C11-C12	1.49	1.49
C11-N4	1.35	1.34
C16-N4	1.36	1.34
C16-C18	1.38	1.38
C18-C3	1.42	1.42
C3-C6	1.42	1.40
C18-C24	1.52	1.51
C24-O9	1.41	1.42
C3-C28	1.45	1.46
C28-N2	1.31	1.29
N2-N7	1.33	1.37

N7-C27	1.40	1.35
C27-S1	1.66	1.70
C27-N19	1.35	1.31
MAE	0.02	

Table S2. Crystallographic and optimized (at B3LYP/6-31+G(d,p) level of theory) bond angles (in °) of PLTSC.

Bond angle	Optimized	Experimental
O22-C6-C3	123.2	124.0
O22-C6-C11	115.9	115.3
C6-C11-N4	117.1	116.9
C6-C11-C12	122.7	123.1
C12-C11-N4	120.2	120.0
C11-N4-C16	124.7	124.9
N4-C16-C18	119.9	120.5
C16-C18-C24	118.7	118.1
C16-C18-C3	119.1	120.1
C18-C24-O9	108.1	111.9
C24-C18-C3	122.2	121.8
C18-C3-C6	118.2	118.8
C18-C3-C28	120.4	119.1
C6-C3-C28	121.4	122.1
C3-C28-N2	121.2	121.4
C28-N2-N7	119.0	114.8
N2-N7-C27	124.3	122.2
N7-C27-S1	118.0	117.5
N7-C27-N19	115.8	119.3
N19-C27-S1	126.2	123.3
MAE	1.3	

Table S3. Second order perturbation theory energies of stabilization interactions (at B3LYP/6-31+G(d,p) level of theory) within structure of PLTSC

Stabilization interaction	Energy [kJ mol^{-1}]
π(S1-C27)→ π(N2-N7)	19
π(C3-C28)→ π(C27-N19)	17
π(N4-C11)→ π(C16-C18)	76
π(C16-C18)→ π(N4-C11)	60
LP(S1)→σ(N7-C27)	53
LP(S1)→σ(N19-C27)	49
LP(N2)→ σ(O22-H23)	93
LP(N7)→π(S1-C27)	212
LP(N7)→π(N2-C27)	176

LP(N19)→π(S1-C27)	274
LP(O22)→ π(C3-C6)	33
π(C6-C11)→ π(C3-C28)	11
π(C6-C18)→ π(C3-C18)	12

Table S4. Crystallographic and optimized (at B3LYP/6-31+G(d,p) level of theory bond lengths (in Å) of Fe-PLTSC.

	Optimized	Crystallographic
fe1-Cl2	2.36	2.27
fe1-Cl3	2.32	2.30
fe1-S4	2.32	2.43
fe1-N10	1.90	2.22
fe1-O5	1.94	1.92
s4-C12	1.68	1.70
C12-N8	1.37	1.31
C12-N9	1.35	1.34
N9-N10	1.38	1.37
N10-C13	1.31	1.30
C13-C15	1.43	1.45
C15-C16	1.45	1.41
C16-O5	1.26	1.30
C16C-C17	1.43	1.42
C17-C18	1.49	1.48
C18-N11	1.34	1.33
N11-C22	1.36	1.37
C22-C24	1.37	1.36
C24-C25	1.51	1.51
C25-O6	1.43	1.43
C24-C15	1.42	1.42
MAE	0.04	

Table S5. Crystallographic and optimized (at B3LYP/6-31+G(d,p) level of theory) selected bond angles (in ^{o}) of Fe-PLTSC.

	Optimized	Crystallographic
Cl2-Fe1-Cl3	98.5	97.7
Cl2-Fe1-S4	91.4	92.0
Cl2-Fe1-N10	173.6	168.5
Cl2-Fe1-O5	89.1	104.2
Cl2-Fe1-O7	79.5	87.0
Fe1-S4-C12	94.8	99.5
S4-C12-N8	123.8	120.5
S4-C12-N9	120.4	122.1
N8-C12-N9	115.7	117.4

C12-N9-N10	120.8	122.0
N9-N10-Fe1	116.8	116.6
Fe1-N10-C13	128.3	128.3
N10-C13-C15	124.2	123.4
C13-C15-C16	121.2	121.8
C13-C15-C24	119.8	118.7
C15-C16-O5	126.0	125.1
O5-C16-C17	116.3	116.4
C16-C17-C18	120.4	122.5
C18-C17-N11	120.3	118.6
C17-N11-C22	124.3	123.9
N11-C22-C24	119.7	119.5
C22-C24-C15	120.0	119.4
C22-C24-C25	117.3	117.5
C24-C25-O	110.0	108.9
MAE	2.1	

Table S6. Crystallographic and optimized (at B3LYP/6-31+G(d,p) level of theory bond lengths (in Å) of Co-PLTSC.

	Optimized	Crystallographic
Co1-S2	2.27	2.21
Co1-S3	2.27	2.22
S2-C21	1.74	1.74
C21-N22	1.36	1.35
C21-N16	1.33	1.32
N16-N12	1.35	1.38
Co-N12	1.94	1.91
N12-C34	1.31	1.30
C34-C33	1.44	1.44
C33-C25	1.43	1.43
C25-C45	1.51	1.51
C45-O15	1.43	1.41
C25-C31	1.38	1.36
C31-N20	1.36	1.35
N20-C36	1.35	1.34
C36-C41	1.49	1.48
C36-C29	1.43	1.41
C29-O14	1.29	1.30
O14-Co1	1.94	1.95
S3-C30	1.74	1.74
C30-N19	1.35	1.34
C30-N17	1.33	1.32

N17-N11	1.35	1.38
N11-Co1	1.94	1.91
N11-C23	1.31	1.30
C23-C6	1.44	1.45
C6-C7	1.44	1.42
C7-O13	1.29	1.31
O13-Co1	1.95	1.97
C7-C10	1.43	1.41
C10-C37	1.49	1.49
C10-N4	1.35	1.34
N4-C8	1.36	1.34
C8-C5	1.38	1.38
C5-C26	1.52	1.51
C26-O18	1.42	1.42
MAE	0.01	

Table S7. Crystallographic and optimized (at B3LYP/6-31+G(d,p) level of theory) selected bond angles (in °) of Co-PLTSC.

	Optimized	Crystallographic
S2-Co1-S3	92.1	92.9
S2-Co1-N12	86.6	85.8
S2-Co1-N11	91.4	94.5
S2-Co1-O13	90.4	89.1
S2-Co1-O14	177.8	177.5
Co1-S2-C21	93.0	94.1
S2-C21-N27	118.9	118.8
S2-C21-N16	125.0	123.4
N22-C21-N16	116.2	117.7
C21-N16-N12	114.9	112.8
N16-N12-Co1	120.5	120.6
Co1-N12-C34	124.8	125.1
N12-C34-C33	126.2	124.3
C34-C33-C25	118.6	118.2
C34-C33-C29	122.4	122.3
C33-C25-C45	122.6	121.9
C25-C45-C15	108.5	109.7
C33-C25-C31	119.8	119.3
C25-C31-N20	119.7	120.1
C31-C20-C36	124.3	123.5
C20-C36-C41	119.7	118.0
C41-C36-C29	121.2	122.2
C36-C29-O14	116.2	118.0

C29-O14-Co1	125.8	121.1
MAE	1.2	

Table S8. Crystallographic and optimized (at B3LYP/6-31+G(d,p) level of theory bond lengths (in Å) of Ni-PLTSC.

	Optimized	Crystallographic
Ni1-S1	2.48	2.43
Ni1-S3	2.48	2.41
S2-C26	1.69	1.69
C26-N10	1.35	1.33
C26-N13	1.37	1.35
N13-N15	1.37	1.39
N15-Ni1	2.10	2.04
N15-C27	1.30	1.30
C27-C29	1.45	1.45
C29-C30	1.43	1.42
C30-C39	1.52	1.51
C39-O5	1.43	1.42
C30-C31	1.38	1.37
C31-N16	1.36	1.36
N16-C33	1.34	1.34
C33-C35	1.49	1.48
C33-C34	1.45	1.44
C34-O4	1.27	1.29
O4-Ni1	2.05	2.05
S23-C42	1.69	1.69
C42-N18	1.35	1.32
C42-N21	1.37	1.36
N21-N23	1.37	1.37
N23-C43	1.30	1.29
C43-C45	1.45	1.46
C45-C46	1.43	1.42
C46-C55	1.52	1.51
C55-O8	1.43	1.41
C46-C47	1.38	1.37
C47-N24	1.36	1.34
N24-C49	1.34	1.34
C49-C51	1.49	1.49
C49-C50	1.45	1.43
C50-O7	1.27	1.30
O7-Ni1	2.05	2.04
MAE	0.02	

Table S9. Crystallographic and optimized (at B3LYP/6-31+G(d,p) level of theory) selected bond angles (in º) of Ni-PLTSC.

	Optimized	Crystallographic
S2-Ni1-S3	93.0	92.5
S2-Ni1-N15	82.7	82.1
S2-Ni1-N23	95.8	102.8
S2-Ni-O4	169.7	168.4
S2-Ni1-O7	90.2	88.8
Ni1-S2-C26	95.2	94.5
S2-C26-N10	121.6	122.7
S2-C26-N13	123.2	122.0
N10-C26-N13	115.1	115.2
C26-N13-N15	122.5	119.8
N13-N15-Ni1	116.3	115.5
N13-N15-C27	115.9	114.9
N15-C27-C29	125.8	122.2
C27-C29-C34	122.4	122.0
C27-C29-C30	117.7	117.6
C29-C34-O4	126.3	125.5
C34-O4-Ni1	129.9	121.9
O4-C34-C33	116.6	117.6
C34-C33-C35	121.0	122.3
C34-C33-N16	119.0	118.9
C33-N16-C31	125.0	124.7
N16-C31-C30	119.5	119.6
C31-C30-C39	117.0	118.4
C30-C39-O5	109.4	112.0
C39-C30-C29	123.4	122.6
MAE	1.6	

Table S10. Crystallographic and optimized (at B3LYP/6-31+G(d,p) level of theory bond lengths (in Å) of Cu-PLTSC.

	Optimized	Crystallographic
Cu1-S2	2.34	2.27
Cu1-O3	2.12	1.89
Cu1-N14	2.15	1.98
Cu1-O35	2.20	2.34
S2-C17	1.69	1.71
C17-N9	1.35	1.32
C17-N12	1.37	1.34
N12-N14	1.36	1.38

N14-C18	1.29	1.29
C18-C20	1.46	1.45
C20-C21	1.45	1.42
C21-O3	1.28	1.30
C21-C22	1.44	1.42
C22-C23	1.49	1.48
C22-N15	1.34	1.33
N15-C27	1.36	1.35
C27-C29	1.38	1.36
C29-C30	1.52	1.51
C29-C20	1.42	1.42
C30-O4	1.43	1.43
MAE	0.04	

Table S11. Crystallographic and optimized (at B3LYP/6-31+G(d,p) level of theory) selected bond angles (in ^{o}) of Cu-PLTSC.

	Optimized	Crystallographic
S2-Cu1-O3	150.4	172.2
S2-Cu1-N14	84.7	86.4
S2-Cu1-O36	116.1	101.0
S2-C17-N9	121.2	121.6
S2-C17-N12	123.9	121.3
N9-C17-N12	114.9	117.1
C17-N12-N14	120.7	119.3
N12-N14-Cu	113.8	116.7
N12-N14-C18	118.4	115.7
N14-C18-C20	123.7	123.5
C18-C20-C21	122.7	122.1
C18-C20-C29	117.5	118.8
C20-C29-C30	121.6	122.5
C29-C30-O4	107.8	108.6
C30-C29-C27	118.5	118.1
C29-C27-N15	118.9	120.4
C27-C15-C22	125.0	123.6
C15-C22-C23	119.2	119.7
C15-C22-C21	119.2	119.5
C22-C21-O3	117.8	116.2
C21-O3-Cu	127.2	127.3
MAE	2.9	

Table S12. Second order perturbation theory energies of selected stabilization interactions (at B3LYP/6-31+G(d,p) level of theory) within structures of investigated complexes

Compound	Stabilization interaction	Energy [kJ mol^{-1}]
Fe-PLTSC	LP(O)→σ(Fe-Cl)	51
	LP(Owater)→LP*(Fe)	355
	π(C-S)→ LP*(Fe)	18
	π(C-N)→ LP*(Fe)	22
Co-PLTSC	LP(S)→σ(O-S)	44
	LP(O)→ σ(Co-O)	25
	σ(Co-N)→σ(Co-N)	29
Ni-PLTSC	LP(S)→σ(Ni-S)	42
	LP(O)→σ(Ni-S)	142
	LP(N)→LP(Ni)	117
Cu-PLTSC	LP(S)→LP(Cu)	87
	LP(O)→LP(Cu)	107
	LP(N)→LP(Cu)	43
	LP(O)→LP(Cu2)	100

Table S13. Binding constant (K_b), the number of binding sites (n) for the competitive measurements with warfarin

Compound	Kb [M^{-1}]	n	R
Fe-PLTSC	2.00×10^5	1.10	0.994
Co-PLTSC	5.25×10^4	1.01	0.983
Ni-PLTSC	6.03×10^6	1.30	0.995
Cu-PLTSC	3.39×10^6	1.20	0.992

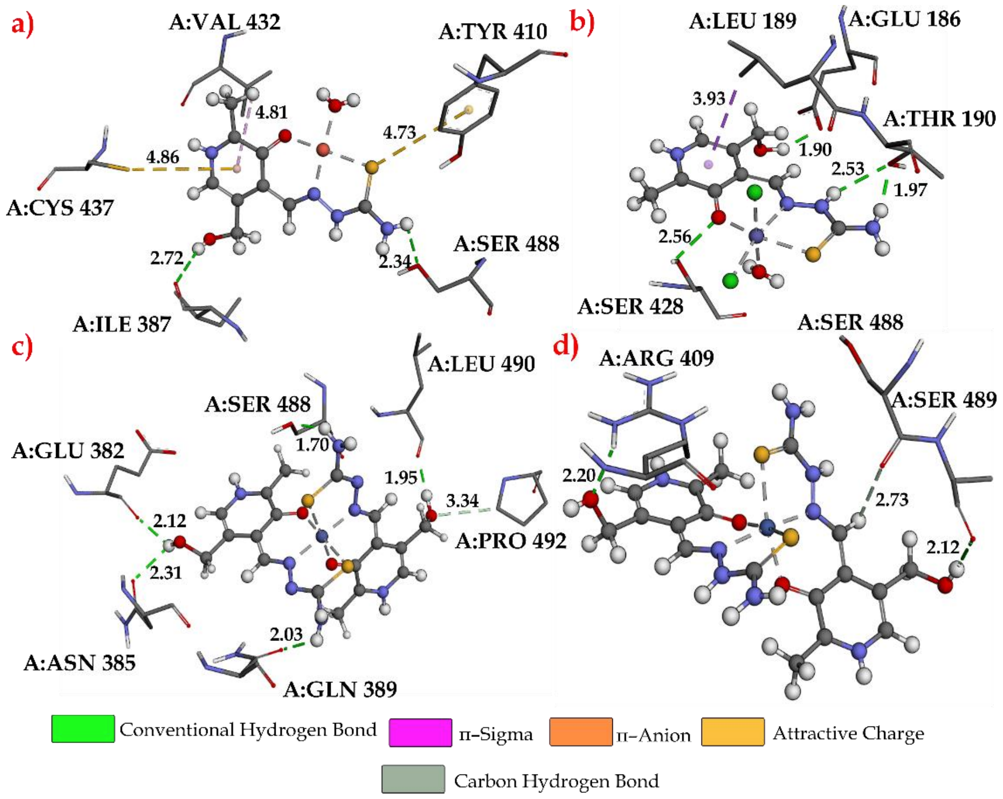

Figure S6. The most stable conformations of investigated complexes in structure of BSA (active pocket IIIA) a) Cu-PLTSC, b) Fe-PLTSC, c) Co-PLTSC, and d) Ni-PLTSC

Table S14. Important thermodynamic parameters (ΔG_{bind} free energy binding, K_i constant of inhibition, ΔG_{total} final total internal energy, ΔG_{tor} torsional free energy, ΔG_{unb} unbound system's energy, ΔG_{elec} electrostatic energy and $\Delta G_{vdw+hbond+desolv}$ is the sum of dispersion and repulsion (ΔG_{vdw}), hydrogen bond (ΔG_{hbond}), and desolvation (ΔG_{desolv}) energy predicted for most stable docking conformations.

Conformations	ΔG_{bind}	K_i (μM)	ΔG_{inter}	$\Delta G_{vdw+hbond+desolv}$	ΔG_{elec}	ΔG_{total}	ΔG_{tor}	ΔG_{unb}
**HSA-Cu-PLTSC-I**	-23.4	78.2	-28.0	-27.1	-0.9	-2.2	4.6	-2.2
**HSA-Cu-PLTSC-II**	-23.5	360.7	-24.2	-23.7	-0.5	-2.2	4.6	-2.2
**HSA-Fe-PLTSC-I**	-23.3	160.4	-26.2	-25.9	-0.3	-1.8	4.6	-1.8
**HSA-Fe-PLTSC-II**	-21.2	0.2	-20.0	-19.2	-0.8	-1.8	4.6	-1.8
**HSA-Co-PLTSC-I**	-24.0	60.6	-30.9	-27.8	-3.1	0.9	6.9	0.9
**HSA-Co-PLTSC-II**	-13.7	0.4	-20.6	-19.2	-1.4	1.1	6.9	1.1
**HSA-Ni-PLTSC-I**	-19.1	438.9	-23.7	-22.2	-1.5	0.3	4.6	0.3
**HSA-Ni-PLTSC-II**	-13.1	0.5	-17.6	-16.8	-0.8	0.1	4.6	0.1

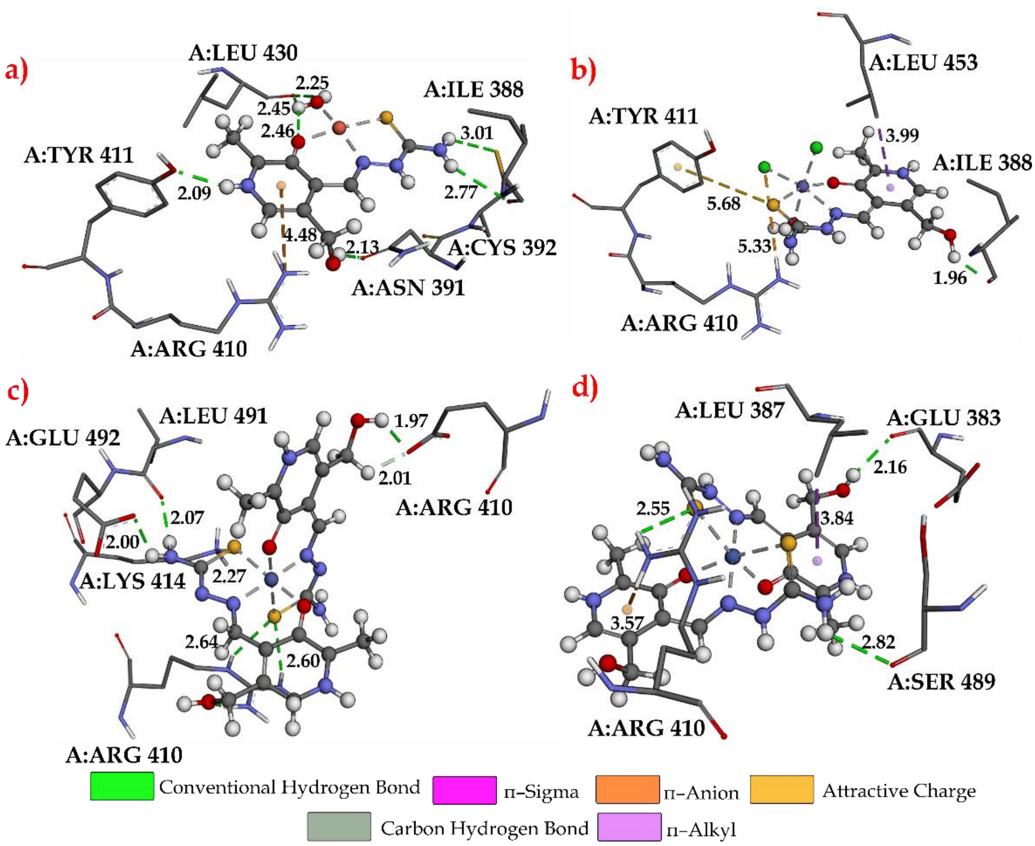

Figure S7. The most stable conformations of investigated complexes in structure of HSA (active pocket IIIA) a) Cu-PLTSC, b) Fe-PLTSC, c) Co-PLTSC, and d) Ni-PLTSC

Table S15. Important thermodynamic parameters (ΔG_{bind} free energy binding, K_i constant of inhibition, ΔG_{total} final total internal energy, ΔG_{tor} torsional free energy, ΔG_{unb} unbound system's energy, ΔG_{elec} electrostatic energy and $\Delta G_{vdw+hbond+desolv}$ is the sum of dispersion and repulsion (ΔG_{vdw}), hydrogen bond (ΔG_{hbond}), and desolvation (ΔG_{desolv}) energy predicted for most stable docking conformations.

Conformations	ΔG_{bind}	K_i (μM)	ΔG_{inter}	$\Delta G_{vdw+hbond+desolv}$	ΔG_{elec}	ΔG_{total}	ΔG_{tor}	ΔG_{unb}
**DNA-Cu-PLTSC-6 base**	-29.3	7.22	-33.9	-33.2	-0.7	-2.2	4.6	-2.2
**DNA-Cu-PLTSC- 10 base**	-31.9	2.50	-36.5	-35.9	-0.6	-2.0	4.6	-2.0
**DNA-Fe-PLTSC-6 base**	-24.0	63.6	-28.5	-27.9	-0.6	-1.2	4.6	-1.2
**DNA-Fe-PLTSC- 10 base**	-26.9	19.3	-31.5	-30.4	-1.1	-1.6	4.6	-1.6
**DNA-Co-PLTSC- 6 base**	-23.3	81.5	-30.2	-30.1	-0.2	0.9	6.9	0.9
**DNA-Co-PLTSC- 10 base**	-26.0	27.3	-32.9	-31.9	-1.0	1.0	6.9	1.0
**DNA-Ni-PLTSC- 6 base**	-25.9	29.0	-30.5	-29.5	-1.0	0.3	4.6	0.3
**DNA-Ni-PLTSC-10 base**	-26.5	22.6	-31.1	-28.6	-2.4	0.1	4.6	0.1

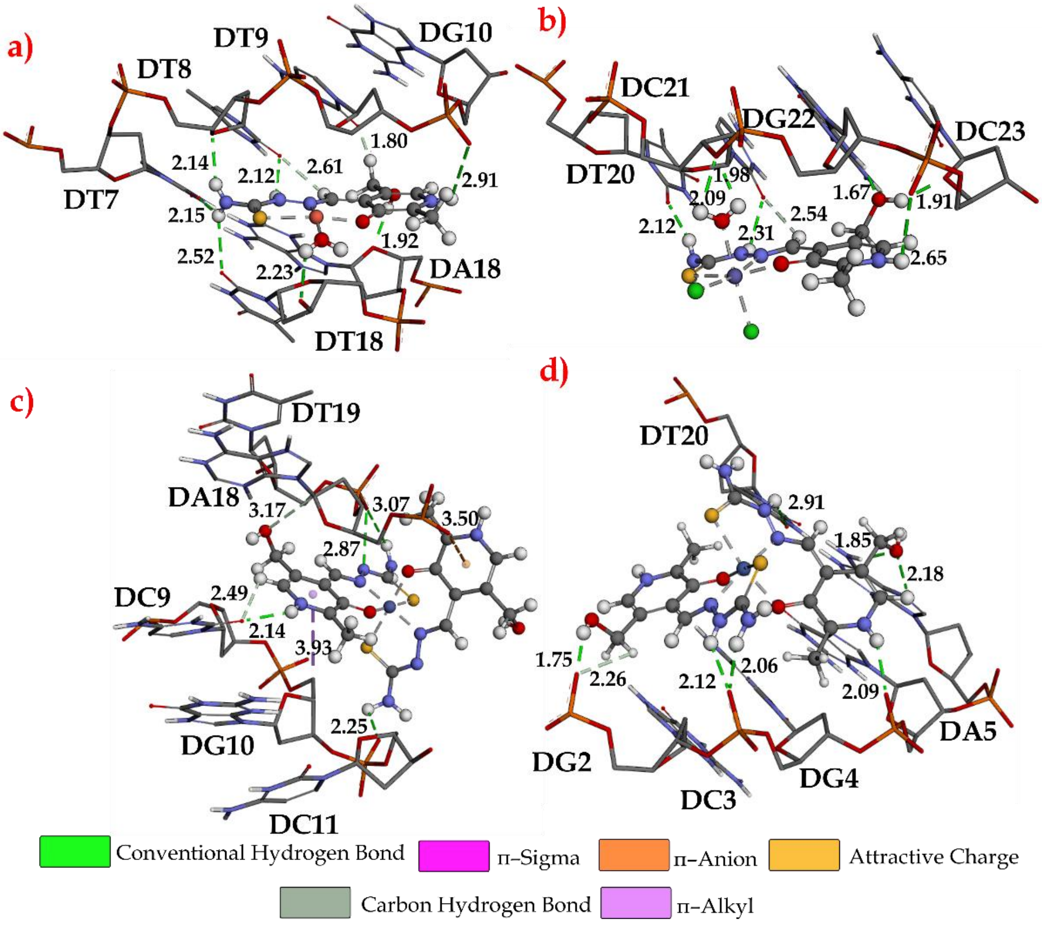

Figure S8. The interactions formed between investigated complexes and ten-base DNA for the most stable conformers: a) Cu-PLTSC, b) Fe-PLTSC, c) Co-PLTSC, and d) Ni-PLTSC.

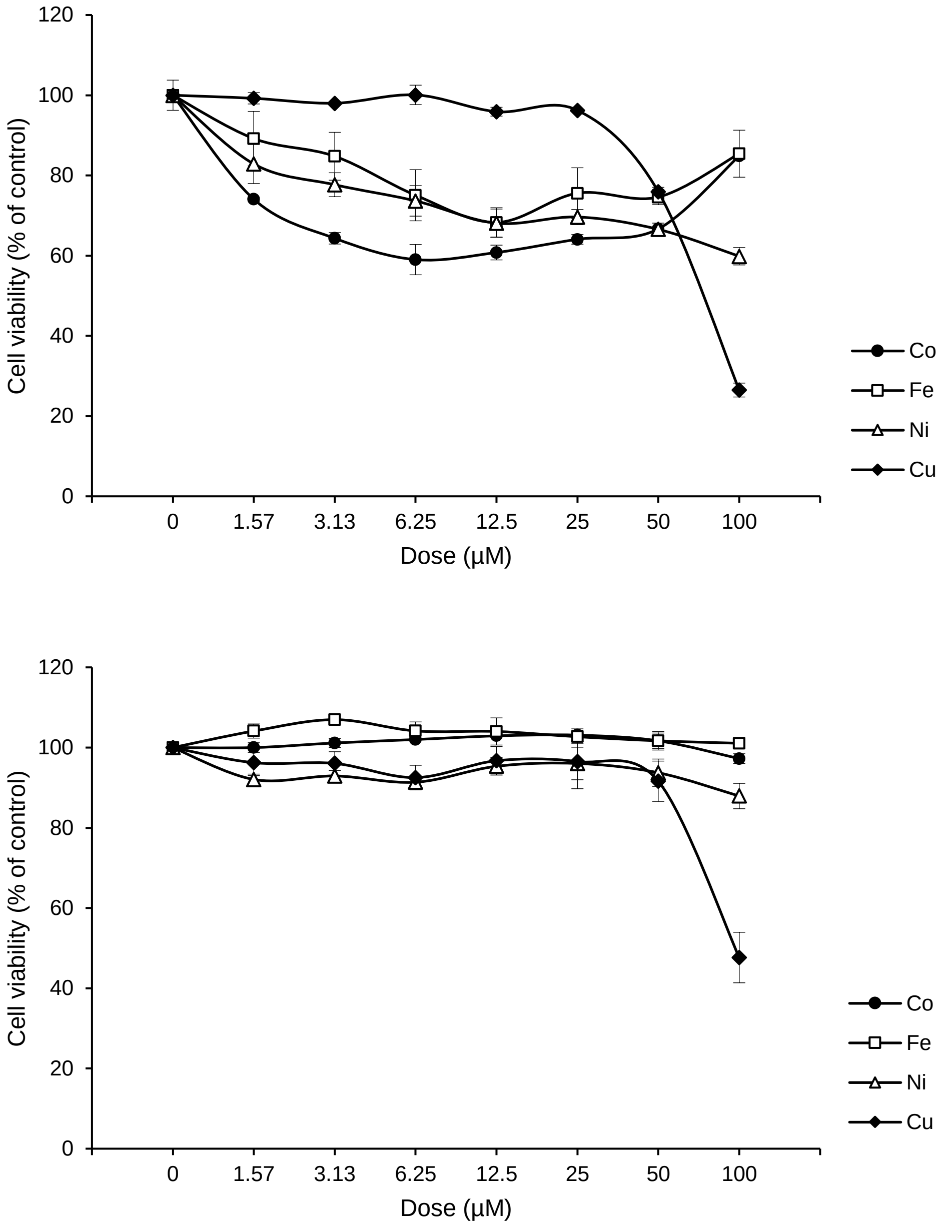

Figure S9. The dose-response curve for the activity towards A375 measured by MTT (upper) and CV (lower) tests.

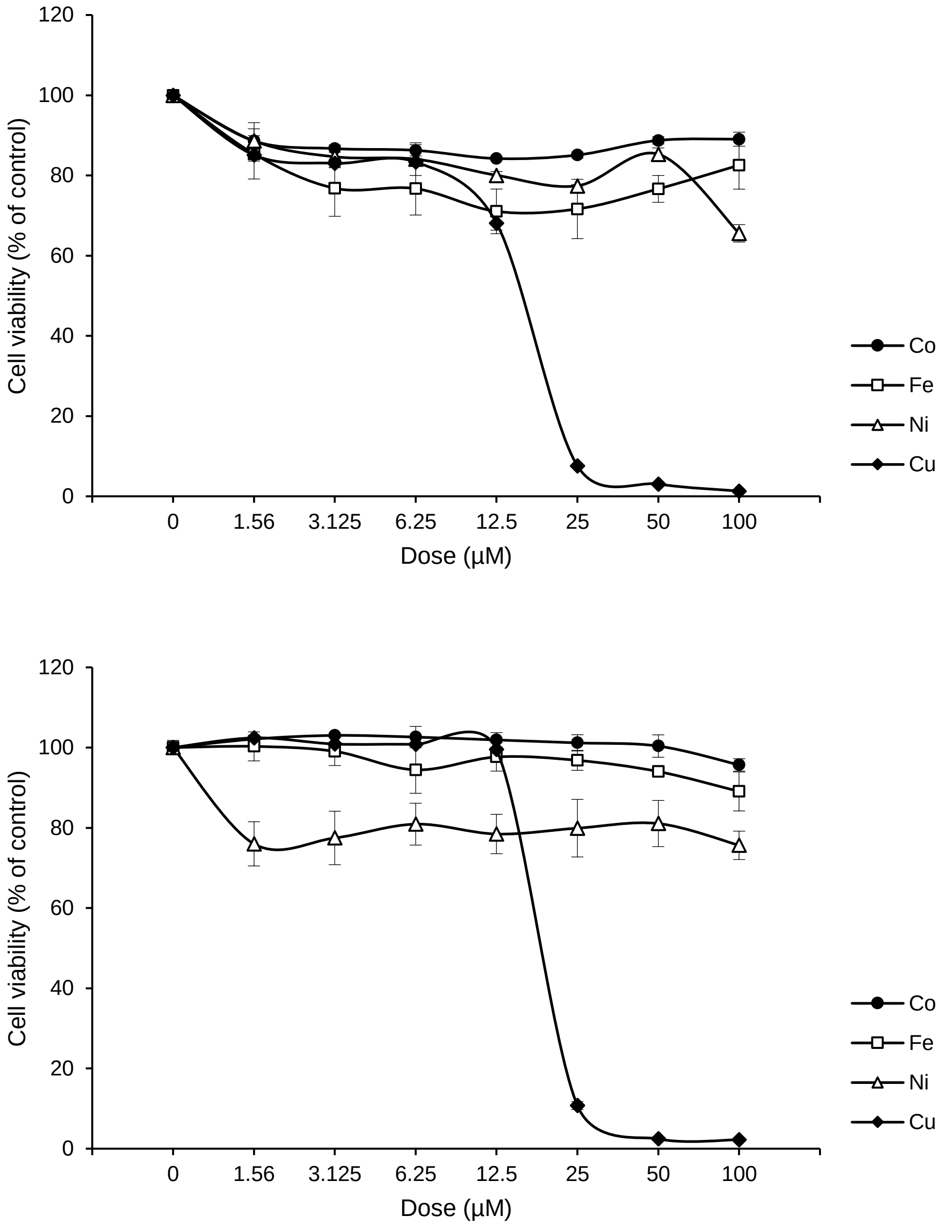

Figure S10. The dose-response curve for the activity towards HCT 116 measured by MTT (upper) and CV (lower) tests.

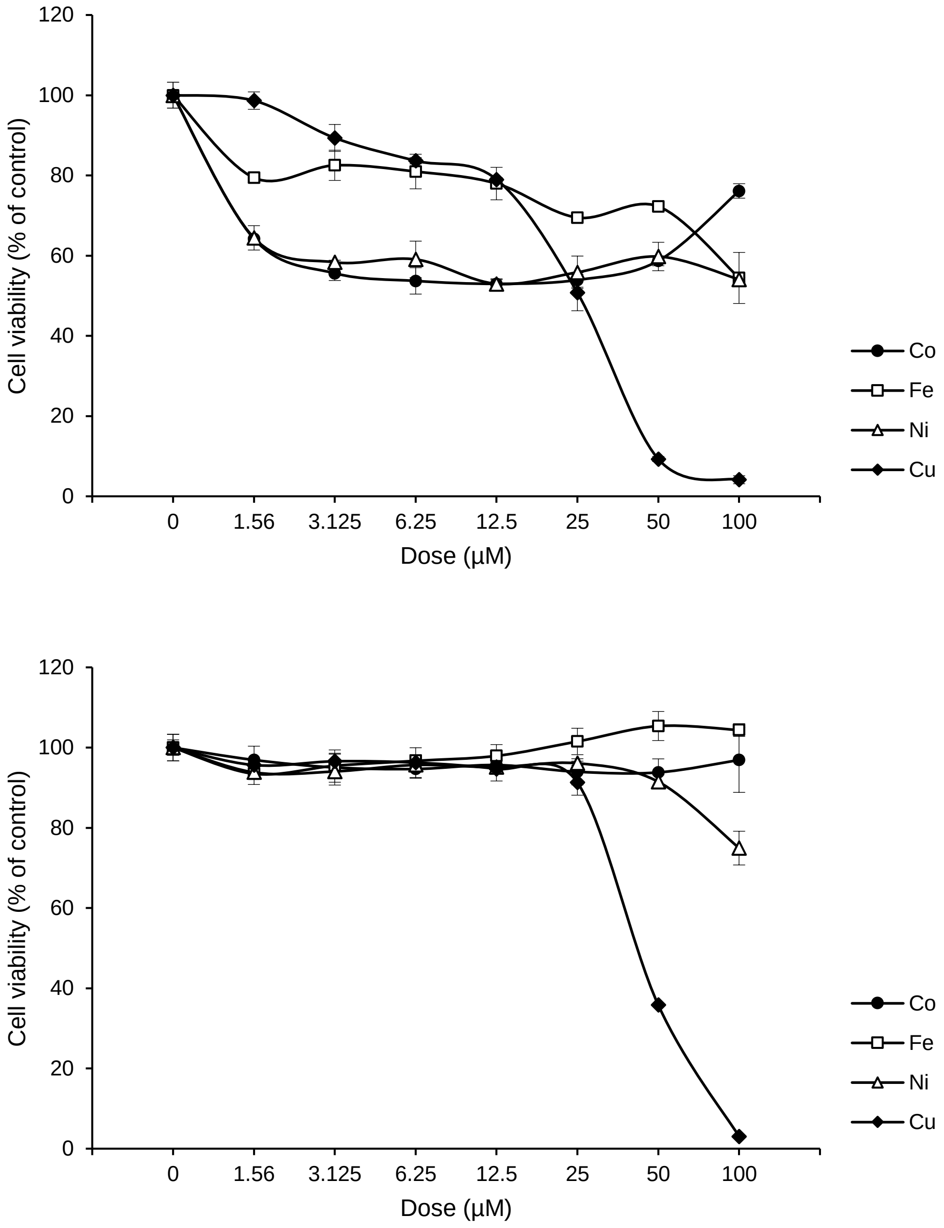

Figure S11. The dose-response curve for the activity towards MCF-7 measured by MTT (upper) and CV (lower) tests.

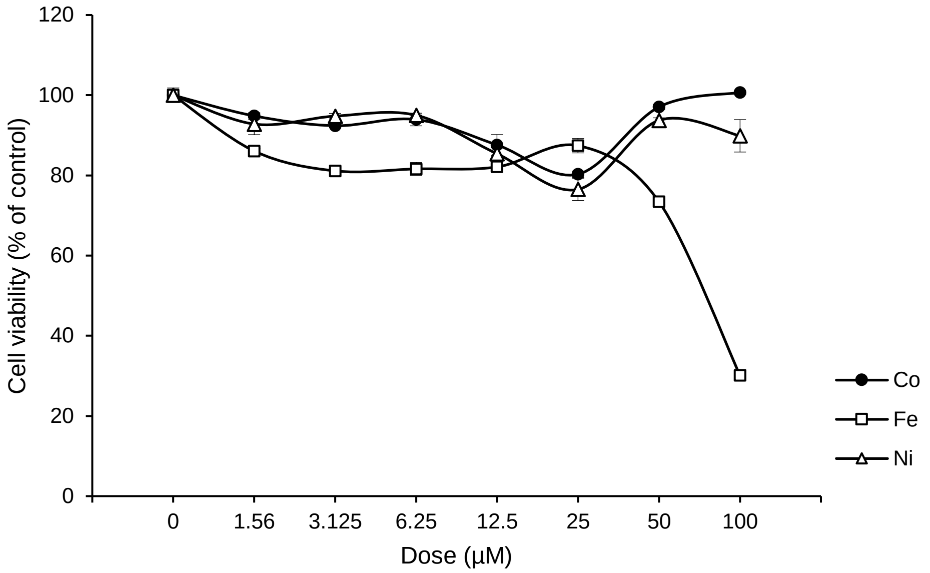

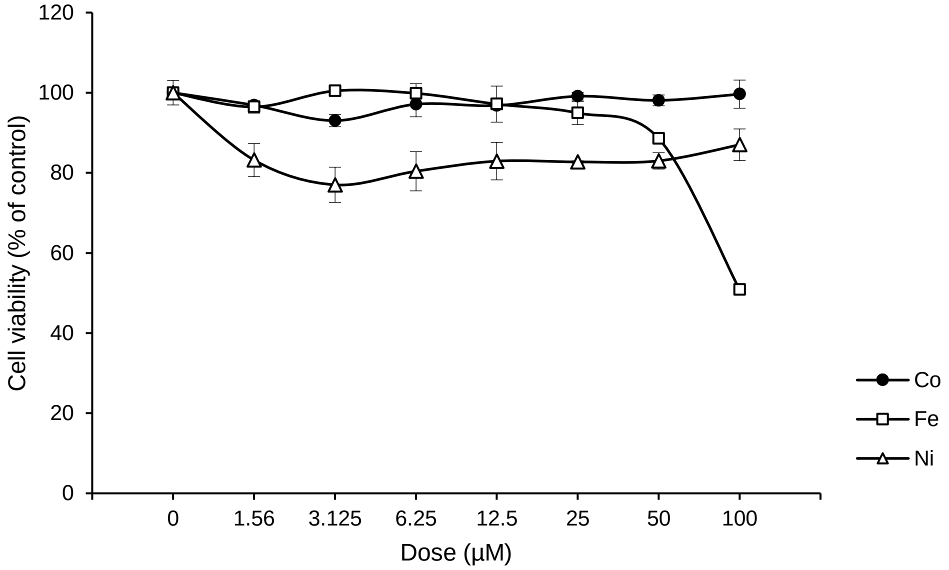

Figure S12. The dose-response curve for the activity of Fe-PLTSC, Co-PLTSC, and Ni-PLTSC towards A2780 measured by MTT (upper) and CV (lower) tests.

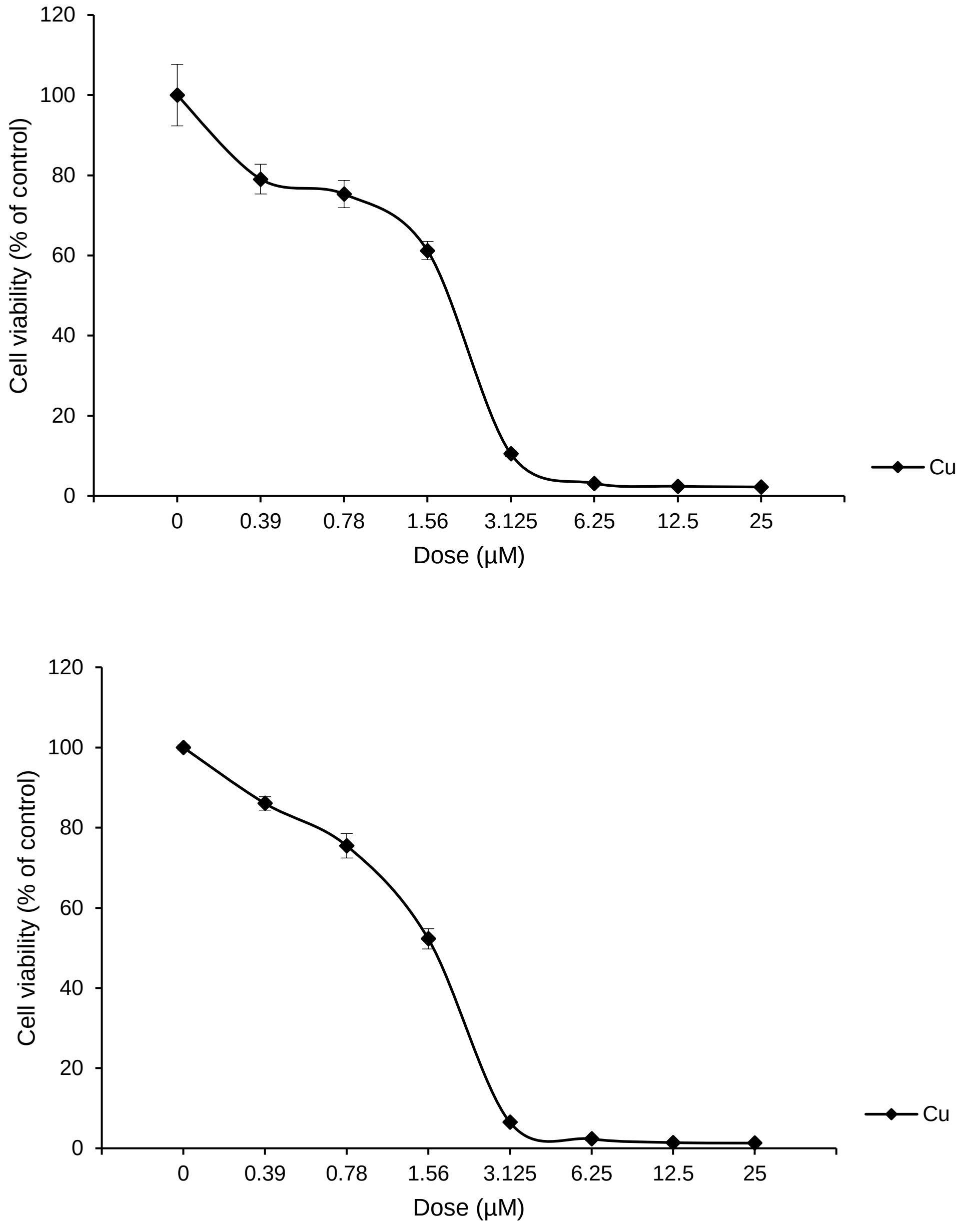

Figure S13. The dose-response curve for the activity of Cu-PLTSC towards A2780 measured by MTT (upper) and CV (lower) tests.

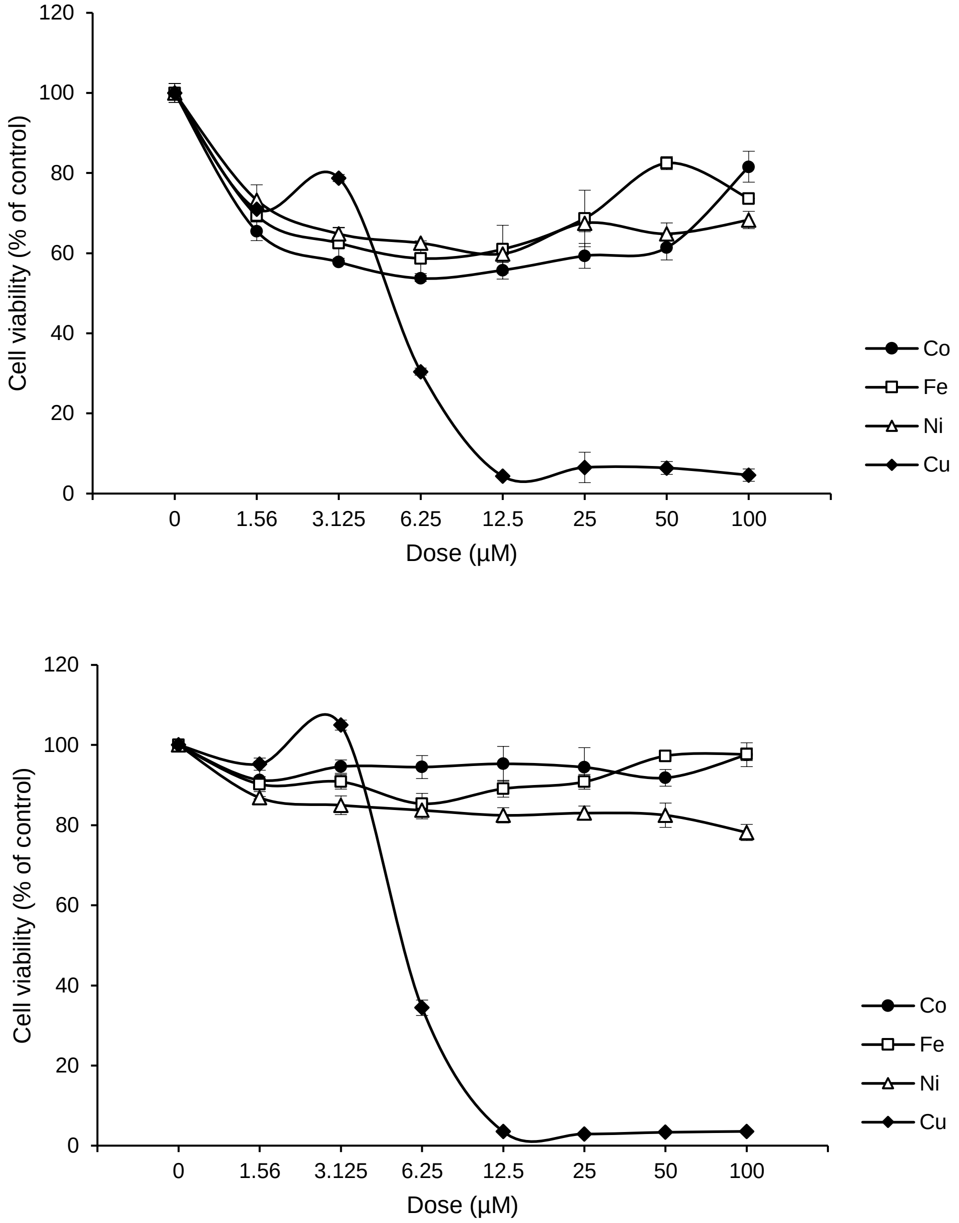

Figure S14. The dose-response curve for the activity towards MRC5 measured by MTT (upper) and CV (lower) tests.
